# Enzyme replacement therapy (ERT) combined with transient low-dose methotrexate (TLD-MTX) results in age- and disease-dependent immune profile changes in Infantile- vs. late-onset Pompe disease patients

**DOI:** 10.3389/fimmu.2026.1843379

**Published:** 2026-09-04

**Authors:** Parisa Amirifar, Ankit K. Desai, Seung-Hye Jung, Sarah P. Young, P. Brian Smith, Cheng Lu, Bijan Abar, Priya S. Kishnani, Trevor D. Burt

**Affiliations:** 1 Division of Medical Genetics, Department of Pediatrics, Duke University Health System, Durham, NC, United States; 2 Biochemical Genetics Laboratory, Duke University Health System, Durham, NC, United States; 3 Division of Neonatology, Department of Pediatrics, Duke University School of Medicine, Durham, NC, United States; 4 Duke Clinical Research Institute, Durham, NC, United States; 5 Children’s Health and Discovery Initiative, Duke University School of Medicine, Durham, NC, United States; 6 Department of Integrative Immunobiology, Duke University School of Medicine, Durham, NC, United States; 7 Duke Molecular Physiology Institute, Durham, NC, United States

**Keywords:** Enzyme replacement therapy (ERT), high and sustained anti-rhGAA IgG antibody titers (HSAT), immune phenotyping, immune profiling, immune tolerance induction (ITI), infantile-onset Pompe disease (IOPD), late-onset Pompe disease (LOPD), methotrexate

## Abstract

**Background:**

Transient low-dose methotrexate (MTX) induces long-term immune tolerance to enzyme replacement therapy (ERT) in Pompe disease; however, the underlying immunological mechanisms remain unclear. We hypothesized that TLD-MTX would reduce immune activation and induce a regulatory phenotype in patients with Infantile-Onset Pompe Disease (IOPD) and Late-Onset Pompe Disease (LOPD) treated with ERT.

**Methods:**

We evaluated immune cell profile changes in patients treated with ERT+MTX using multiparameter flow cytometry on peripheral blood mononuclear cells collected at baseline and six months post-treatment. We analyzed 40 immune parameters in (1) thirteen unpaired samples from ERT+MTX-untreated (n=10) and -treated (n=3) IOPD patients and (2) six paired pre- and post-treatment samples from LOPD (n=6) patients. Linear regression analyses were used to assess correlations between immune parameters and disease activity measured by urinary glucose tetrasaccharide (Glc_4_; a breakdown product of glycogen).

**Results:**

In IOPD, ERT+MTX-treated samples (median age: 12.3 months) showed reduced % CD16^+^ monocytes and increased classical CD14^+^CD16^−^ monocytes, suggesting a shift toward a less activated immune profile. Untreated samples had higher % mature memory B cells and lower % plasmablasts, central memory CD4^+^ and CD8^+^ T cells, and TIGIT^+^ CD8^+^ T cells. In contrast, LOPD patients (median age: 51.5 years) exhibited decreased % B cells and increased % TIGIT^+^ CD4^+^ T cells after treatment. Baseline Glc_4_ levels were higher in IOPD than LOPD (42.5 vs. 7) and decreased in both groups following ERT+MTX. In untreated IOPD patients, Glc_4_ levels positively correlated with CD16^+^ monocytes, mature memory B cells, and terminally differentiated CD8^+^ T cells; these associations were not observed after treatment. In LOPD, T follicular regulatory cells correlated with Glc_4_ prior to treatment but not after, and no additional significant correlations were identified.

**Discussion:**

These findings demonstrate distinct immune profiles before and after ERT+MTX treatment in IOPD and LOPD patients. In IOPD, reduced pro-inflammatory monocytes and memory T cells provide evidence to suggest decreased immune activation. In LOPD, increased TIGIT^+^ CD4^+^ T cells may reflect a regulatory mechanism contributing to immune tolerance.

## 1 Introduction

Pompe disease, also known as glycogen storage disease type II (OMIM 232300), is an autosomal recessive lysosomal disorder caused by a deficiency of the enzyme acid α-glucosidase (GAA) (1). This enzyme is essential for breaking down glycogen within lysosomes, and its deficiency leads to glycogen accumulation in cardiac, skeletal, and smooth muscle, resulting in progressive muscle weakness (2). Pompe disease is broadly classified into two subtypes: infantile-onset Pompe disease (IOPD) and late-onset Pompe disease (LOPD), differentiated by age at symptom onset and the presence or absence of cardiomyopathy in the first year of life. IOPD, the most severe form, involves minimal GAA activity, rapid glycogen buildup, and severe symptoms like hypotonia, cardiomyopathy, and respiratory failure, often leading to death within two years without treatment. Patients with LOPD progress more slowly than IOPD, with symptom onset ranging from infancy to late adulthood (3, 4).

Enzyme replacement therapy (ERT) with recombinant human acid α-glucosidase (rhGAA; alglucosidase alfa) has altered the natural history of Pompe disease; however, the response to ERT remains variable due to factors such as the age at treatment initiation, underlying pathology, dose of ERT, and cross-reactive immunologic material (CRIM) status (5, 6). CRIM represents residual expression of GAA, which can be detected using standard immunological assays. CRIM-negative patients, lacking expression of native GAA, and a subset of CRIM-positive patients can develop high and sustained anti-rhGAA IgG antibody titers (HSAT; titers of ≥ 1:12,800), reducing ERT efficacy (7, 8). Immune tolerance induction (ITI) strategies, including a short course of rituximab, methotrexate, and IVIG, have been developed to mitigate the immune response to rhGAA in CRIM-negative and CRIM-positive patients, mainly when initiated early in ERT-naïve patients, and this has become a standard approach in such cases (9). However, rituximab induces broad and prolonged B cell depletion and is not widely accessible. Consequently, a novel approach using transient low-dose methotrexate (TLD-MTX) was implemented and proved successful in this population (10). This approach may offer a more manageable and widely available option for controlling anti-rhGAA antibody production in Pompe patients, with potentially fewer immunogenic side effects.

The mechanism underlying tolerance induced by TLD-MTX is not fully understood; however, several preclinical studies have investigated the potential pathways involved. Preclinical studies in GAA knock-out mice have shown that TLD-MTX used concomitantly with ERT, reduces IgG antibodies against ERT via a mechanism involving IL-10^+^, TGF-β^+^, and Foxp3^+^ regulatory B cells (11, 12). A related study identified erythropoiesis up-regulation and found that methotrexate temporarily reduced mature RBCs in blood and immature RBCs in bone marrow, with increased immature, nucleated RBCs in spleen and blood during ITI, suggesting a role of erythropoiesis in this process (13).

In a previous study, we evaluated ITI using TLD-MTX co-initiated with rhGAA in treatment-naïve, infantile-onset Pompe disease (IOPD) patients. The TLD-MTX ITI protocol demonstrated feasibility and safety, with no serious adverse events and good overall tolerance (10). However, no immune phenotyping studies have been conducted to characterize unique immunological features in IOPD and LOPD patients undergoing initiation of ERT, particularly those treated with TLD-MTX. Recently, we identified potential immunophenotypic signatures in patients who developed HSAT against ERT with rhGAA (14). The results of that study suggested that changes in both T and B cells significantly impact HSAT development, indicating the potential benefit of adding T-cell-targeted therapies alongside B-cell depletion strategies like rituximab. Moreover, evidence pointed to innate immune activation playing a pivotal role in HSAT pathogenesis, emphasizing the need for detailed, comprehensive immune profiling to advance treatment strategies in Pompe disease.

In this study, we investigated the potential effects of TLD-MTX on immune status by analyzing immune phenotyping data from IOPD and LOPD patients who received TLD-MTX in an ERT-naïve setting. This is the first study to evaluate immune modulation with TLD-MTX in Pompe disease patients. To assess immune profile changes following ERT+MTX treatment, we examined key immune cell subsets, including CD4^+^ and CD8^+^ T cells, B cells, NK cells, and myeloid cells (e.g., monocytes and dendritic cells) in both cohorts. Additionally, we evaluated the correlation between Glc_4_, a biomarker of glycogen storage, and the patients’ immune profiles. We hypothesized that ITI with TLD-MTX would reduce immune activation and induce a regulatory phenotype in patients with IOPD and LOPD treated with ERT.

## 2 Materials and methods

### 2.1 Study population

For the LOPD cohort, participants were included if they met the following criteria: 1) the presence of two confirmed pathogenic variants in the GAA gene (15) and, 2) current treatment with ERT in combination with TLD-MTX.

For the IOPD cohort, inclusion was based on 1) a confirmed diagnosis of IOPD, characterized by significantly reduced or absent GAA enzyme activity, the presence of two pathogenic GAA variants, and cardiomyopathy developing within the first year of life (4), and 2) treatment with ERT in combination with TLD-MTX. Due to the unavailability of matched pre-ERT samples from patients treated with TLD-MTX, we included age-matched ERT-naïve IOPD patients as unpaired controls.

For both cohorts, the study required the availability of sufficient blood samples and detailed clinical data for inclusion as well as the collection of samples approximately six months after the initiation of ERT combined with TLD-MTX.

### 2.2 Data collection

Clinical and laboratory data including *GAA* variants, CRIM status, age at ERT initiation, duration of ERT at the time of sample collection, and longitudinal data on anti-rhGAA IgG antibody titers, urinary glucose tetrasaccharide (Glc_4_), aspartate aminotransferase (AST), and alanine aminotransferase (ALT) levels were extracted from medical records using Duke Health’s REDCap and Maestro Care (Epic) applications. As of January 2025, baseline and available follow-up data spanning up to three years after ERT combined with MTX treatment were collected.

CRIM status was assigned based on predicted status of known GAA variants, as previously described (16). Anti-rhGAA IgG antibody titers were measured by Sanofi or LabCorp, as previously described (17). Quantitative analysis of urinary glucose tetrasaccharide (Glc_4_) was carried out using liquid chromatography-tandem mass spectrometry (LC-MS/MS) in the Biochemical Genetics Laboratory at Duke University Hospital (18).

### 2.3 Sample preparation and immune profiling

Peripheral blood was collected via venipuncture into acid-citrate-dextrose tubes (BD Vacutainer, Franklin Lake, NJ). Plasma and peripheral blood mononuclear cells (PBMCs) were separated using Ficoll density gradient centrifugation (GE Healthcare, Uppsala, Sweden). The plasma layer was first isolated, aliquoted into 1 mL portions, and stored at −80 °C. PBMCs were washed, counted, and re-suspended in a solution of 90% FBS (Gemini, West Sacramento, CA) and 10% DMSO (Sigma-Aldrich, St. Louis, MO) solution, then gradually cooled to −80 °C using a Cool Cell freezing container. The following day, the cells were transferred to vapor phase liquid nitrogen for long-term storage.

The Duke Immune Profiling Core (DIPC) performed cell preparation, staining, and flow cytometry analysis following previously established protocols (14). Antibodies used for flow cytometry, detailed in **Supplementary Table 1**, were sourced from BioLegend (San Diego, CA) and BD Biosciences.

### 2.4 Statistical analysis

Statistical analysis was performed using Stata, R, and GraphPad Prism (version 10.0). The Mann–Whitney U test was used to compare variables between pre- and post-ERT+MTX groups in unpaired IOPD patients. For paired LOPD patients, differences between pre- and post-ERT+MTX measurements were assessed using the Wilcoxon matched-pairs signed-rank test. Simple linear regression was performed to evaluate associations between urinary Glc_4_ levels and immune parameters in both IOPD and LOPD patients. In addition, multiple linear regression analyses adjusted for age were used to assess the relationship between Glc_4_ levels and immune parameters in both cohorts. Regression coefficients (β) with 95% confidence intervals (CI) and p-values are reported, and a P-value of <0.05 was considered statistically significant for all analyses.

### 2.5 Study approval

Written informed consent was obtained from all participants or their parents or legally authorized representatives for all participants included in the study. All patients were enrolled under Duke Institutional Review Board (IRB)-approved study protocols: Pro00001562 (Determination of Cross-Reactive Immunological Material [CRIM] Status and Longitudinal Follow-up of Individuals with Pompe disease), Pro00010830 (A Long-Term Follow-Up Study of Pompe Disease), Pro00007612 (Genetic Disease Repository for Blood, Urine, and Tissue), and/or Pro00051144 (Identify alterations in phenotypically defined PBMC subpopulations and potential generation of anti-Myozyme cellular reactivities in Pompe disease patients following alglucosidase alfa^®^, [Lumizyme^®^] therapy).

## 3 Results

### 3.1 Patient cohorts and treatment regimen

The treatment regimen for immune tolerance induction using transient low dose (TLD) methotrexate (MTX) consists of three cycles of three daily doses of MTX either SC or oral (0.4 mg/kg per day) given every other week or every week starting with the first dose of rhGAA (20 mg/kg), which is also given every other week or every week (see **Supplementary Figure 4**). While MTX is given only for a total of 9 doses over the first 3 doses of ERT for immune tolerance induction, patients continue to receive ERT with rhGAA every other week or every week (10).

#### 3.1.1 LOPD

The LOPD cohort included six patients, predominantly male (5 males and one female). Notably, all LOPD patients carried the c.-32-13T>G variant on one allele, a common pathogenic variant associated with a less severe disease phenotype (**Table 1**). However, the second allele carried more severe pathogenic variants in several cases.

**Table 1:** Demographic characteristics of the IOPD and LOPD patients.

Patients ID	Gender	GAA variants ^1^		Age at sample^2^	Time on ERT+MTX at sample (M)	ERT dose^3^ (mg/kg)	Peak titer	Time on ERT at peak titer (M)	The latest titer	Time on ERT at the latest titer (M)
		Allele 1	Allele 2							
LOPD Cohort										
MTX-LOPD1	M	c.-32-13T>G ^a^	c.877G>A ^b^	63.09	6.44	20 EOW	800	3	400	36
MTX-LOPD2	M	c.-32-13T>G ^a^	c.525del ^d^	38.63	8.98	20 EOW	1600	12	800	24
MTX-LOPD3	M	c.-32-13T>G ^a^	c.2481 + 102_2646 + 31del ^d^	63.73	10.82	20 EOW	6400	18, 30	3200	36
MTX-LOPD4	M	c.-32-13T>G^a^	c.1655T>C ^b^	77.89	5.75	20 EOW	6400	6, 24	3200	36
MTX-LOPD5	F	c.-32-13T>G ^a^	c.525del ^d^	31.67	5.06	20 EOW	51200	12	6400	32
MTX-LOPD6	M	c.-32-13T>G ^a^	c.258dup ^d^	32.17	4.83	20 EOW	0	–	400	27
IOPD Cohort										
Naïve-IOPD1*	F	c.2238G>A ^d^	c.2560C>T ^d^	6.3	0.0	–	–	–	–	
Naïve-IOPD2	M	c.1441T>C ^b^	c.2550C>T ^d^	3.2	0.0	–	–	–	–	
Naïve-IOPD3	M	c.1979G>A ^b^	c.2560C>T ^d^	3.1	0.0	–	–	–	–	
Naïve-IOPD4*	F	c.2560C>T ^d^	c.2560C>T ^d^	6.6	0.0	–	–	–	–	
Naïve-IOPD5	F	c.1437 + 1G>A ^d^	c.2227C>T ^d^	1.2	0.0	–	–	–	–	
Naïve-IOPD6	F	c.953T>C ^b^	c.2481 + 102_2646 + 31del ^d^	1.4	0.0	–	–	–	–	
Naïve-IOPD7*	M	c.379_380del ^d^	c.2269C>T ^d^	2.8	0.0	–	–	–	–	
Naïve-IOPD8	M	c.525delT ^d^	c.655G>A ^b^	11.6	0.0	–	–	–	–	
Naïve-IOPD9	F	c.670C>T ^c^	c.799_803delinsA ^d^	4.3	0.0	–	–	–	–	
Naïve-IOPD10	M	c.307T>G ^b^	c.525del ^d^	4.2	0.0	–	–	–	–	
MTX-IOPD1 ^4^	M	c.1457 + 2T>C ^d^	c.665T>G ^b^	3.4	4.7	20 W	200	3	0	14
MTX-IOPD2 ^5^	F	c.525del ^d^	c.1979G>A^b^	13.0	9.2	20 EOW	25600	6	6400	9
MTX-IOPD3	M	c.706del ^e^	c.1805C>T ^b^	16.0	7.2	20 EOW, with the frequency increased to weekly after 2 months	6400	3	3200	5

1 GAA Allele Severity Classification Based on Kroos et al., 2008 (62): ^a^Potentially mild, ^b^Potentially less severe, ^c^Less severe, ^d^Very severe, ^e^Unknown, ^2^Age is presented in years for LOPD patients and in months for IOPD patients, ^3^All IOPD and LOPD patients received Lumizyme, ^4^TLD-MTX protocol for LOPD patients consists 9 doses of oral MTX at 10 mg for LOPD patients and 9 doses of oral MTX at 2.5 mg for IOPD, except 2.5 mg for first 3 dose of MTX, followed by 1.25 mg for remaining doses in MTX-IOPD1, ^5^first MTX dose of 3rd cycle was skipped in MTX-IOPD2.

*IOPD patients with CRIM-negative.

CRIM, cross-reactive immunologic material; EOW, every other week; W, weekly; ERT, enzyme replacement therapy; TLD-MTX, transient low-dose methotrexate.

Paired samples were collected from each patient before and after initiation of ERT combined with TLD-MTX (henceforth referred to as ERT+MTX). The pre-ERT+MTX samples were obtained at a median age of 50.9 years (range: 31.3–77.7 years), and post-ERT+MTX samples were collected at a median age of 51.5 years (range: 32.0–78.1 years), with an average interval of 6.1 months (4.8–10.8 months) after the initiation of treatment.

#### 3.1.2 IOPD

The IOPD cohort consists of children who were newly diagnosed with Pompe Disease and were followed at by providers at geographically diverse sites, with ability to collect limited blood due to blood volume issues, also the challenge as there is so much from a clinical perspective to address including the diagnosis and discussions of immune modulation, all these present a challenge of obtaining pre- and post-treatment samples from the same patients. For this reason, and distinct from the LOPD group, the IOPD cohort analysis was carried out on samples from treatment naïve (i.e., untreated) patients and other unmatched patients whose samples were obtained after treatment. A single baseline sample was obtained from ten ERT-naïve IOPD patients (i.e., pre-ERT) at a median age of 4.5 months (range: 1.1–11.6 months), while samples were collected from three unique (i.e., unmatched) patients after treatment with ERT+MTX (i.e., post-ERT+MTX) at a median age of 12.3 months (range: 8.0–16.4 months), approximately 7.2 months (4.7–9.2 months) after starting treatment. The IOPD cohort included thirteen patients in total.

Within the IOPD cohort, the untreated group comprised five females and five males (3 CRIM-negative and 7 CRIM-positive), while the ERT+MTX-treated group included one female and two males (all CRIM-positive) (**Table 1**). The majority of patients carried at least one very severe mutation, such as c.2560C>T or c.525del, both of which are associated with aggressive disease phenotypes (**Table 1**). In all the cases, the variants identified have been noted in IOPD.

#### 3.1.3 Clinical outcome and laboratory result

**Table 1** summarizes the demographic characteristics of six LOPD and three IOPD patients who received methotrexate alongside enzyme replacement therapy. The lifetime average ERT dose for these patients is shown in **Supplementary Figure 1**. Ten ERT-naïve IOPD patients who served as the pre-treatment comparison group are also included in this table.

#### 3.1.4 Clinical Characteristics of the IOPD cohort

In our study, none of the IOPD patients receiving ERT+MTX required invasive ventilatory support. MTX-IOPD1, MTX-IOPD2, and MTX-IOPD3 initiated therapy at 3.4, 3.9, and 6.6 months of age, respectively, with a mean age at treatment initiation of 4.6 months. At baseline, patient MTX-IOPD1 required continuous positive airway pressure (CPAP) support, which continued until three months after ERT initiation (alglucosidase alfa, 20 mg/kg every other week). After 15.5 months of ERT, this patient transitioned to nighttime bilevel positive airway pressure (BiPAP) and reported no respiratory issues at a follow-up visit 52.9 months post-ERT. Patient MTX-IOPD2 initially required non-invasive positive pressure ventilation (PPV) but unfortunately passed away at 15 months of age due to respiratory arrest secondary to a viral infection during flu season. Patient MTX-IOPD3 discontinued nighttime BiPAP support six months after starting ERT, indicating respiratory improvement.

Monitoring key biomarkers CK, AST, and Glc_4_ over 36 months for LOPD patients and over 12 months for IOPD patients revealed fluctuations across individual cases (**Supplementary Table 2**) (**Supplementary Figure 2**). Regarding CK levels, in patients MTX-IOPD1 and MTX-IOPD2, the levels increased twice the baseline several months after treatment began and rarely dropped below the baseline. For patient MTX-IOPD3, follow-up data was unavailable (**Supplementary Figure 2A**).

Regarding aspartate aminotransferase (AST) levels, both patients MTX-IOPD1 and MTX-IOPD2 showed an increase one month after treatment initiation. While AST levels in MTX-IOPD1 remained variable over time, patient MTX-IOPD2 exhibited a sustained elevation, reaching a 1.5-fold increase from baseline by 12 months post-treatment (age:15.9 months) (**Supplementary Figure 2B**). For patient MTX-IOPD3, only baseline and 12-month post-treatment AST levels (age: 18.6 months) were available, and these showed a decrease.

**Figure 1:**
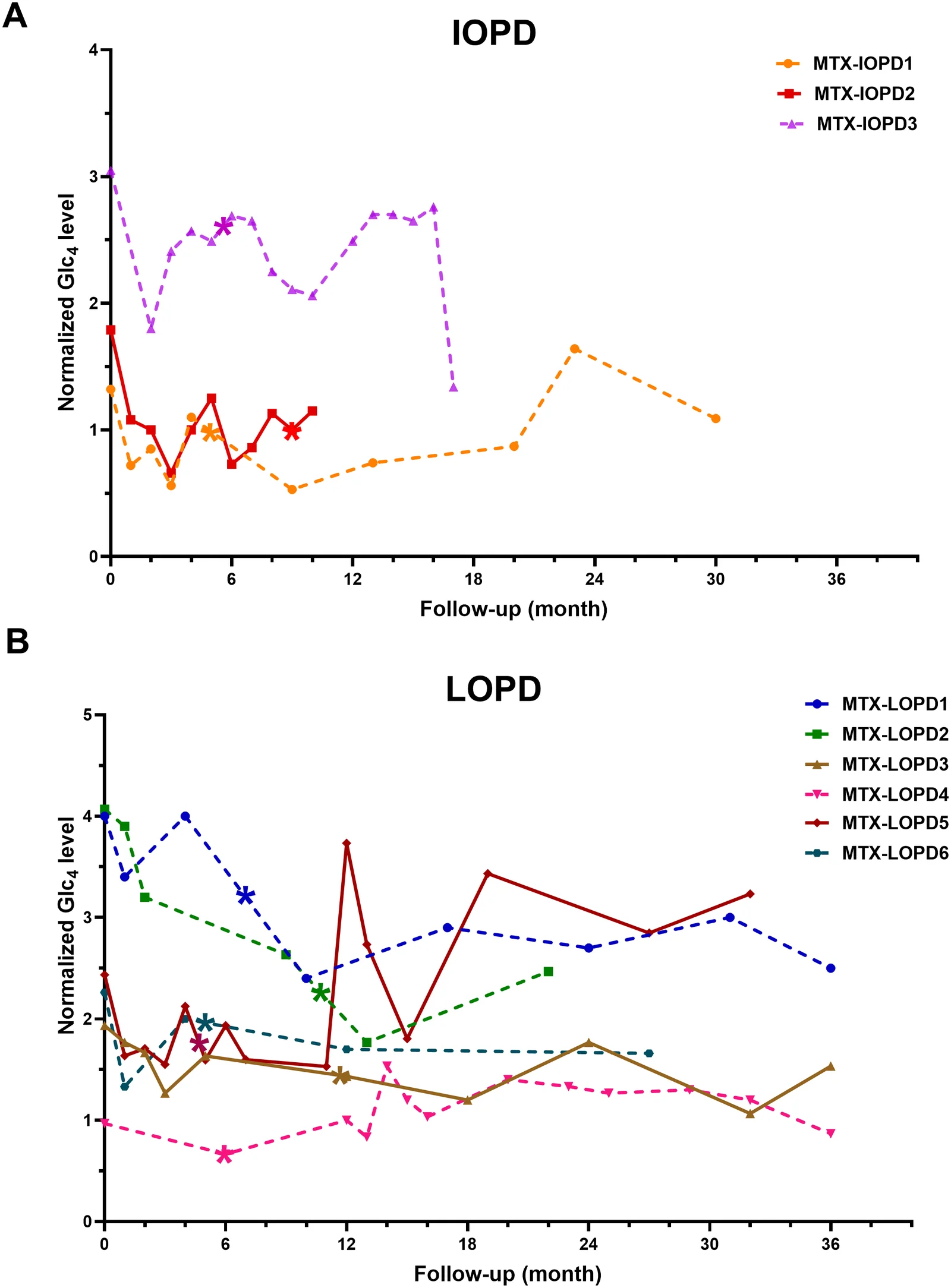
Normalized Glc_4_ levels (expressed as a ratio to age-specific reference ranges) in immune-tolerant and high antibody titer patients receiving ERT combined with TLD-MTX. **(A)** Patients with IOPD. **(B)** Patients with LOPD. The solid line represents patients with high Ab titers, while the dashed line indicates patients with low Ab titers. The asterisk (*) indicates the time points at which samples were collected for immune profiling after initiation of ERT.

All three IOPD patients demonstrated a sharp decline in Glc_4_ levels within the first 3 months of therapy, indicating that enzyme replacement therapy is initially effective in reducing glycogen accumulation (**Figure 1**). However, variability in Glc_4_ levels after 3 months suggests that the sustained response to treatment may not be consistent across all IOPD patients, especially when on dose of 20 mg/kg every 2 weeks. However, Glc_4_ levels did not return to the pre-treatment baseline, except in patient MTX-IOPD1, who exhibited a transient increase above baseline at 24 months, followed by a subsequent reduction. This fluctuation was observed only once during the 32-month follow-up period.

#### 3.1.5 Clinical characteristics of the LOPD cohort

In the LOPD cohort, the average age of symptom onset was 26 years (range: 15–45 years), while the average age at diagnosis was 50 years (range: 30–77 years). All patients experienced muscle weakness and gait difficulties. At baseline, four patients (MTX-LOPD1, MTX-LOPD2, MTX-LOPD3, and MTX-LOPD6) required nighttime non-invasive ventilatory support. These patients reported stabilization and, in some cases, symptom improvement following ERT initiation. Patients MTX-LOPD4 and MTX-LOPD5 initiated nighttime BiPAP at 6 and 18 months after starting ERT, respectively, with subsequent improvement in symptoms.

No infusion-related reactions were reported among LOPD patients except MTX-LOPD5. Approximately one year after beginning ERT, MTX-LOPD5 experienced a heavy feeling in the chest, sensations of warmth, and a severe headache during an infusion. These symptoms were successfully managed with pre-medications, including solumedrol, diphenhydramine, and acetaminophen, allowing the patient to continue ERT without further adverse events.

As shown in **Figure 1**, Glc_4_ levels in the LOPD cohort showed a downward trend. During the 36 months following ERT+MTX therapy, Glc_4_ levels did not return to baseline levels except in patients MTX-LOPD4 and MTX-LOPD5 whose Glc_4_ levels increased to 1.5 times baseline after 18 and 12 months of treatment, respectively. CK, AST, and ALT levels were stable in LOPD patients during 36 months of follow-up (**Supplementary Table 2**).

### 3.2 Immune phenotyping

Multiparameter flow cytometry was performed using cryopreserved peripheral blood mononuclear cells (PBMCs) that were isolated from peripheral blood samples of all patients. One blood sample was analyzed from each IOPD patient (i.e., 10 untreated and 3 ERT+MTX treated patients), while 2 paired (i.e., one pre-ERT+MTX and one post-ERT+MTX) samples were analyzed from each of the LOPD patients. Two multicolor panels were used, as previously described (14), to identify and measure the frequency of detailed cell subsets, including cells involved in innate (e.g., monocyte, dendritic cell, NK cells) and adaptive (e.g., T cell, B cell) immune responses (**Supplementary Table 1**). Measured cell frequencies were compared between pre- and post-ERT+MTX treatment groups within the IOPD and LOPD cohorts separately, and significant differences were observed in several immune cell phenotype parameters after initiation of ERT+MTX (**Table 2**).

**Table 2:** Summary of immunophenotyping parameters in LOPD and IOPD patients.

Parameter	IOPD				LOPD			
	ERT+MTX-untreated (n=10)		ERT+MTX-treated (n=3)		Pre-ERT+MTX (n=6)		Post-ERT+MTX (n=6)	
	Mean	SD	Mean	SD	Mean	SD	Mean	SD
Age at sample	4.5 months	3.07	12.3 months	4.2	50.9 years	19.5	51.5 years	19.6
Length of ERT at time of sample	0	–	7.08 months	2.25	0	–	7.5 months	2.6
Monocyte Parameters								
All CD14^+^ Monocytes	63.534	21.12	81.33	5.50	88.51	3.03	87.75	3.48
Classical Monocytes (%monocytes)	88.66	6.53	98.23	1.68	93.91	4.68	93.40	5.54
Non-Classical Monocytes	8.44	6.19	1.58	1.72	5.79	4.65	6.10	5.44
Dendritic cells parameters								
All Dendritic Cells (DCs)	15.66	12.70	6.54	1.46	4.16	2.11	4.34	1.33
Myeloid Dendritic Cells (mDC; % DCs)	45.64	20.42	46.1	7.23	71.46	10.22	68.58	12.50
Plasmacytoid Dendritic Cells (pDC; % DCs)	11.06	8.75	4.92	2.47	8.19	6.52	9.49	10.85
All NK cells	4.44	3.24	2.08	1.07	12.42	8.78	12.52	11.38
CD16^+^CD56^+^ NK cells (% NK cells)	48.9	16.39	40.96	24.27	62.91	16.22	72.83	20.14
CD16^+^CD56^++^ NK cells	2.55	0.93	1.82	1.73	2.62	3.50	1.58	0.64
CD16^-^CD56^++^ NK cells	17.81	10.98	19.3	8.60	8.52	8.2	7.28	4.52
B cell parameters								
All CD19^+^ B cells	12.53	5.88	18.06	10.38	14.32	6.97	9.77	5.37
Memory B cells (% B cells)	1.61	1.26	3.95	1.42	12.88	7.38	15.32	10.19
Plasmablasts (% IgD^-^ B cells)	11.68	8.31	2.48	2.04	2.22	1.61	5.38	5.87
Naive B cells	94.08	4.13	91.220	3.62	72.81	12.63	66.65	22.36
Transitional B cells	19.85	14.63	9.33	6.47	2.5	0.51	3.82	2.37
Unswitched memory B cells	0.74	0.46	1.60	0.77	5.14	4.47	4.71	4.30
T cell parameters								
All CD3^+^ T cells	60.66	22.29	74.63	11.86	65.36	17.08	71.71	14.70
All CD4^+^ T cells	75.93	6.52	76.36	11.53	71.91	10.08	74.16	8.81
CD4+ Central Memory	13.12	5.45	6.86	0.93	27.35	19.85	25.86	18.27
CD4^+^Naive	85.83	5.59	89.96	4.27	62.83	23.30	64.51	18.33
CD4^+^Terminal Effector	0.27	0.23	1.47	1.85	2.42	2.82	3.37	4.35
CD4^+^ Effector	0.78	0.62	1.77	1.61	7.71	5.51	6.49	4.13
CD4^+^ PD-1^+^	0.41	0.34	0.3	0.17	0.98	0.24	1.15	0.44
CD4^+^ TIGIT^+^	0.73	0.46	0.41	0.34	1.69	0.68	2.26	0.67
CD4^+^ CD38hi	96.7	2.48	94.16	2.95	56.58	16.18	54.86	10.38
CD4^+^ Activated (HLADR+CD38hi; %CD4^+^ T cells)	0.55	0.35	0.66	0.37	1.06	0.50	0.87	0.37
CD4^+^ Tfh Cells	0.38	0.30	0.46	0.61	2.12	2.72	2.57	2.52
CD4^+^ Tfr Cells (%CD25^+^ CD127^low^ TFh cells)	7.68	3.79	3.35	1.28	2.75	1.42	2.32	0.72
Treg (CD25^+^ CD127^low^)	3.52	1.84	3.38	1.83	3.28	0.78	3.64	0.77
CD4^+^ Th17	0.92	0.85	3.28	3.81	2.84	3.64	4.37	4.16
CD4^+^ Th1	25.67	10.07	19.53	2.82	28.65	9.13	31.11	9.04
CD4^+^ Th2	72.91	10.23	76.40	6.50	67.25	12.01	62.51	11.46
All CD8^+^ T cells	15.06	7.04	18.23	7.57	21.23	8.07	20.2	7.16
CD8^+^ Central Memory	11.09	7.09	2.10	0.77	2.20	1.46	2.64	2.28
CD8^+^ Naive	83.11	7.97	92.56	1.33	55.13	21.51	52.11	22.79
CD8^+^ TEMRA	1.59	1.30	3.06	1.78	22.16	14.54	25.08	15.42
CD8^+^ Effector	4.20	5.04	2.28	0.72	20.55	16.88	20.17	19.22
CD8^+^ PD-1^+^	0.55	0.33	0.67	0.52	4.79	3.54	6.27	5.56
CD8^+^ TIGIT^+^	2.25	1.38	0.49	0.21	10.82	9.06	13.77	11.40

#### 3.2.1 Immune phenotyping of IOPD cohort

##### 3.2.1.1 Lower CD16^+^ monocytes and higher CD14^+^CD16^-^ monocytes are observed in IOPD patients treated with ERT+MTX

**Figure 2:**
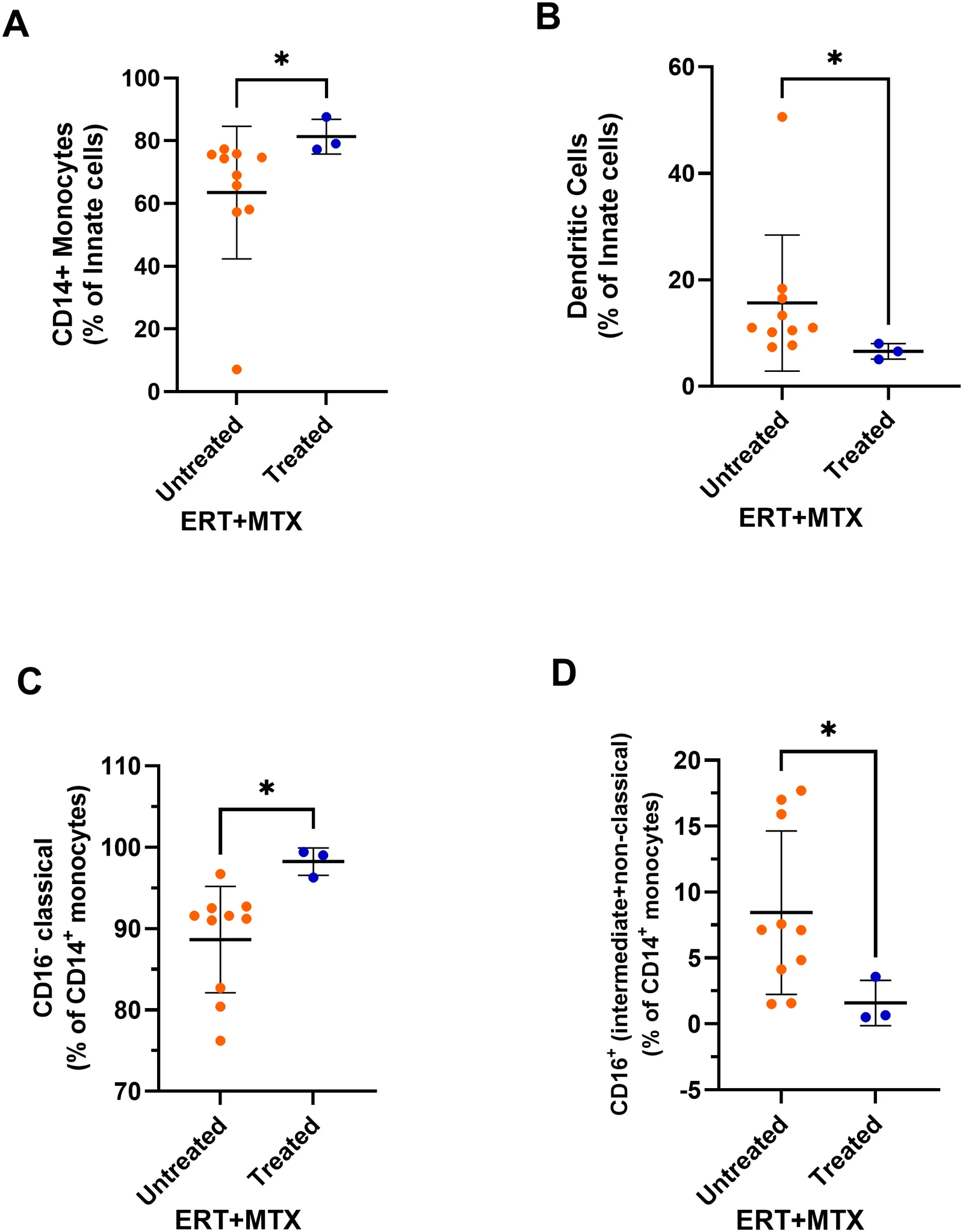
Alterations in monocytes and dendritic cells in IOPD patients after ERT+MTX treatment. Comparison of innate immune cell populations in IOPD patients before and after ERT+MTX treatment. Shown are **(A)** CD14^+^ monocytes (% of innate cells), **(B)** dendritic cells (% of innate cells), and monocyte subsets expressed as a percentage of CD14^+^ monocytes, including **(C)** classical monocytes (CD16^-^ CD14^+^) and **(D)** CD16^+^ monocytes (intermediate + non-classical). Untreated samples are shown in orange, and ERT+MTX-treated samples in blue. Statistical comparisons were performed using the Mann-Whitney test. *P < 0.05, line represents median.

Using standard flow cytometry gating, we detected a relatively higher percentage of all CD14^+^ monocytes (81.33% vs 63.53 of innate cell gate, P value 0.018) and a relative decrease in HLA-DR^+^CD14^-^ dendritic cells (6.54% vs. 15.66 of innate cell gate, P value=0.028) in the ERT+MTX treated IOPD group compared to ERT+MTX untreated IOPD group (**Figures 2A, B**). We then measured the relative frequency of classical (CD14^+^CD16^−^) monocytes in relation to CD16^+^ monocytes, which include both intermediate (CD14^+^CD16^+^) and non-classical (CD14^low/-^CD16^+^) monocytes (see gating in **Supplementary Figure 3A**) (19). We found that there was a significantly lower mean percentage of CD16^+^ (i.e., non-classical and intermediate) monocytes (1.58 vs. 8.44% of CD14^+^ monocytes, P value= 0.028) and a relatively increased mean percentage of CD14^+^CD16^-^ classical monocytes (98.23 vs. 88.66% of CD14^+^ monocytes, P = 0.014) in the ERT+MTX treated IOPD group compared to ERT+MTX untreated IOPD group (**Figures 2C, D**).

##### 3.2.1.2 Higher mature memory B cells and lower plasmablasts were observed in IOPD patients treated with ERT+MTX

**Figure 3:**
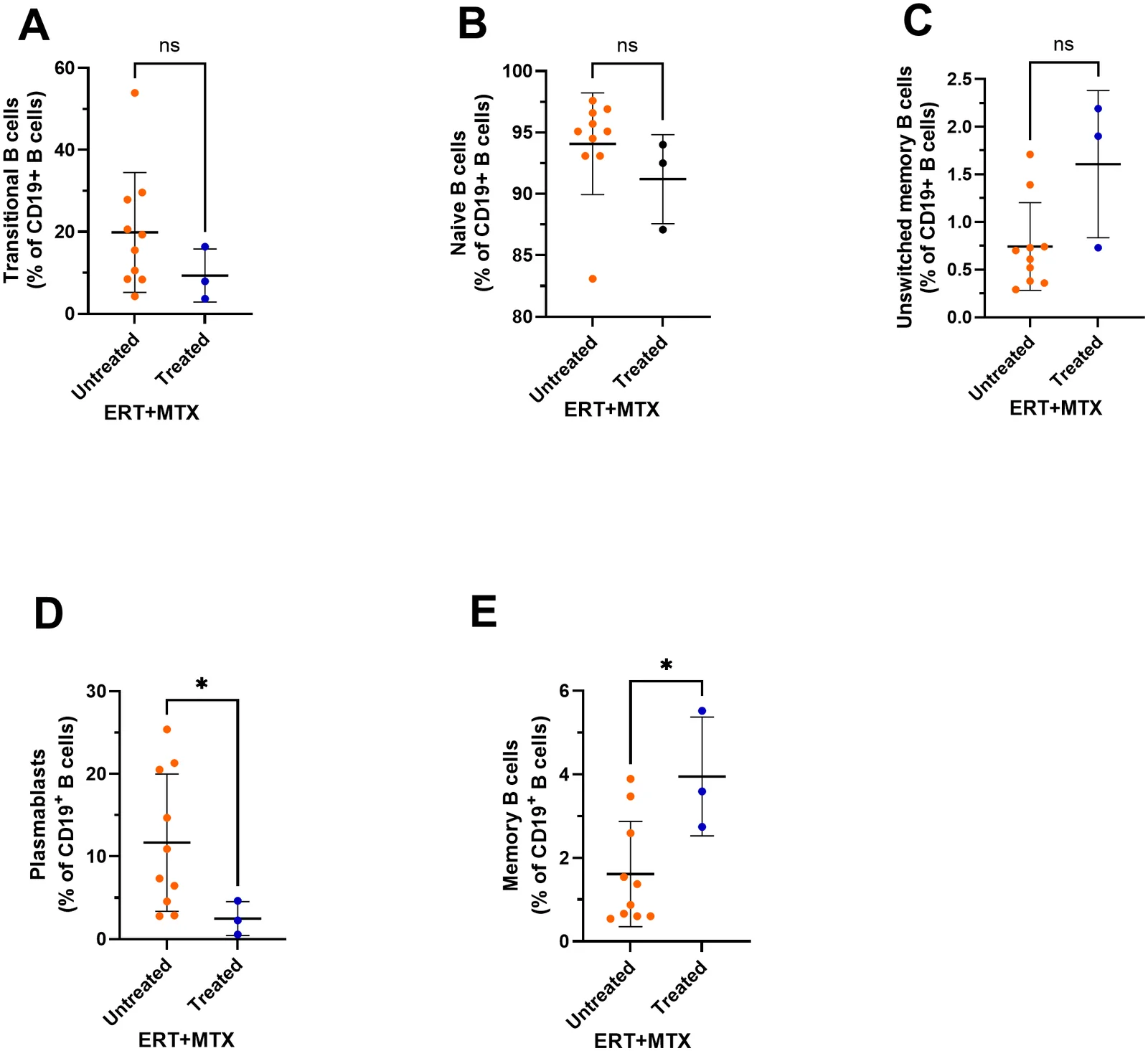
Reduction in immature plasma cells in the ERT+MTX-treated group of IOPD patients. Comparison of antibody-secreting B cell subpopulations (as percent of CD19^+^ B cells) in IOPD patients at baseline and post-ERT+MTX treatment, including: **(A)** transitional B cells (CD19^+^CD20^+^CD38hi CD24hi), **(B)** naïve B cells (CD19^+^IgD^+^CD27^-^), **(C)** unswitched memory B cells (CD19^+^IgD^+^CD27^+^), **(D)** memory B cells (CD19^+^IgD^-^CD27^+^), and **(E)** plasmablasts (CD19^+^CD27^+^IgD^-^CD20^-^CD38^++^). ERT+MTX-untreated samples are represented in orange, and ERT+MTX-treated samples in blue. Statistical comparisons were performed using the Mann-Whitney test. *P < 0.05, “ns” indicates a non-significant difference, line represents median.

To assess whether there were any differences in circulating B cell subsets between ERT+MTX untreated and treated IOPD patients, we analyzed the percentages of various B cell maturation states in PBMCs. This included transitional B cells (the earliest B cells transitioning from the bone marrow to the periphery; identified as CD19^+^CD20^+^CD38^++^CD24^++^), naïve B cells (antigen-specific but not yet activated; CD19^+^IgD^+^CD27^-^), unswitched memory B cells (previously activated in response to an antigen but have not undergone germinal center reactions or class-switching of the IgG heavy chain, thought to correspond to splenic marginal zone B cells; CD19^+^IgD^+^CD27^+^), switched memory B cells (previously activated and matured via IgG heavy chain class-switching; CD19^+^IgD^-^CD27^+^), and plasmablasts (early antibody-secreting cells CD19^+^CD27^+^IgD^-^CD20^-^CD38^++^) (**Figure 3**). We found that the ERT+MTX treated IOPD group had a markedly lower percentage of plasmablasts compared to ERT+MTX untreated IOPD patients, with a nearly fivefold decrease (2.48% vs. 11.68% of IgD- B cells; P = 0.049) (**Figure 3D**). This was accompanied by an increase in mature memory B cells in ERT+MTX treated IOPD patients, which was approximately 2.5 times higher than in ERT+MTX untreated IOPD patients (3.95% vs. 1.61% of B cells; P = 0.045) (**Figure 3E**). Additional B cell subsets were not significantly different between the ERT+MTX vs. non-treated group (see **Figure 3**, **Supplementary Table 3**).

##### 3.2.1.3 Lower central memory CD4^+^ and CD8^+^ T cells in ERT+MTX treated IOPD patients

**Figure 4:**
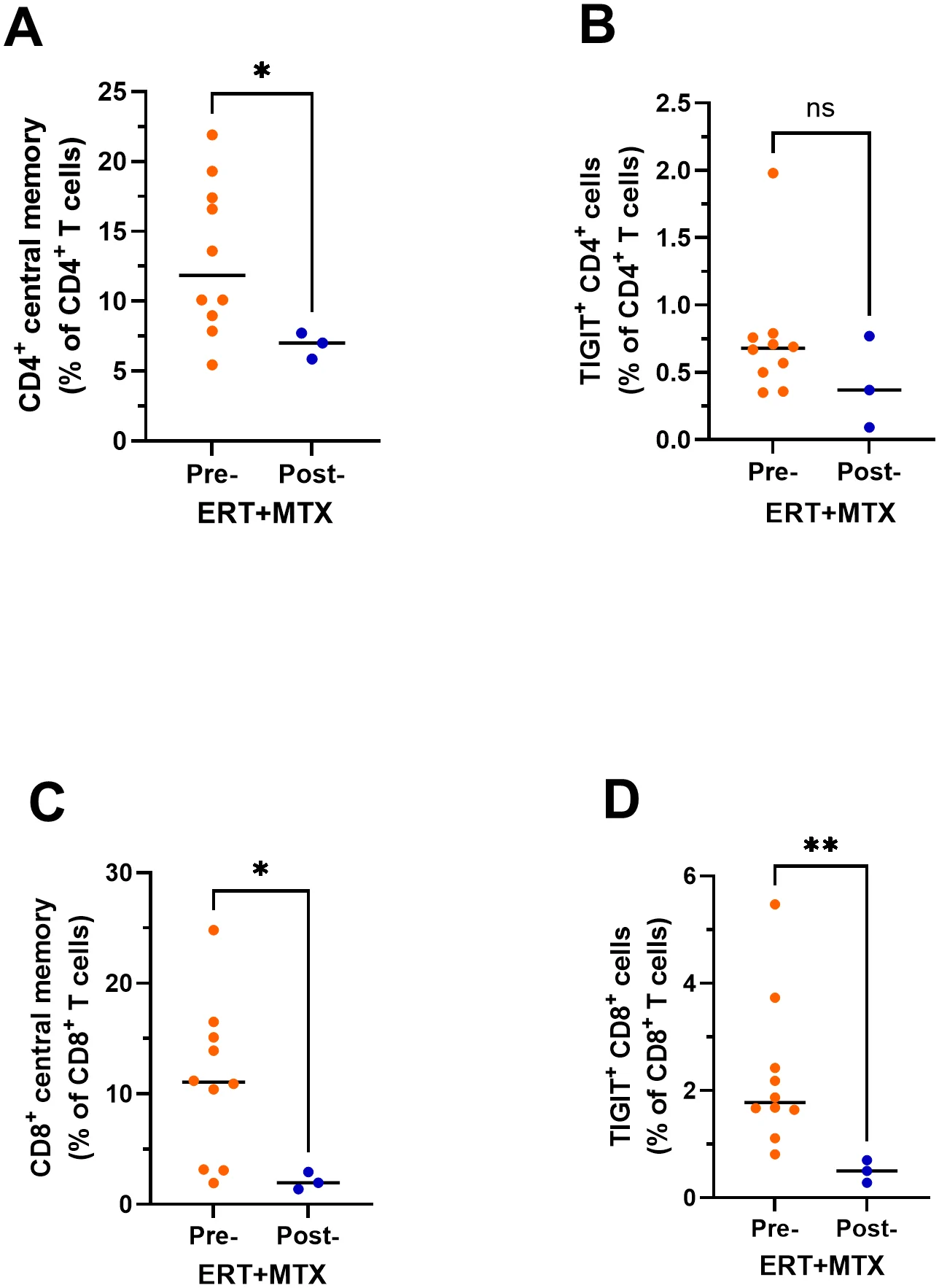
Alterations in T cell subsets in IOPD patients following ERT+MTX treatment. Comparison of T cell subpopulations (as a percent of CD4^+^ or CD8^+^ T cells) in IOPD patients at baseline and post-ERT+MTX treatment, including **(A)** CD4^+^ central memory T cells, **(B)** TIGIT^+^ CD4^+^ T cells, **(C)** CD8^+^ central memory T cells, and **(D)** TIGIT^+^ CD8^+^ T cells. ERT+MTX-untreated samples are represented in orange, and ERT+MTX-treated samples in blue. Statistical comparisons were performed using the Mann-Whitney test. *P < 0.05, **P < 0.01; “ns” indicates a non-significant difference, line represents median.

We assessed the maturation states of CD4^+^ and CD8^+^ T cells using specific markers for various subsets: Naïve (CD45RA^+^CCR7^+^), Central Memory (CD45RA^-^CCR7^+^), Effector/Effector Memory (CD45RA^-^CCR7^-^), and CD45RA^+^ Terminal Effector-Memory (TEMRA; CD45RA^+^CCR7^-^). Our analysis demonstrated that the frequency of central memory CD4^+^ T cells in the ERT+MTX treated IOPD group was just over half that of the ERT+MTX untreated IOPD group (6.86% vs. 13.12% of CD4^+^ T cells; P = 0.042) (**Figure 4A**). Similarly, the frequency of central memory CD8^+^ T cells in the ERT+MTX treated was less than 1/5 that of the untreated IOPD group (2.10% vs. 11.09% of CD8^+^ T cells; P = 0.028) (**Figure 4C**). Additional CD4^+^ and CD8^+^ maturation subsets, including naïve, effector memory, and TEMRA, were not significantly different between the groups (**Table 2**; **Supplementary Table 3**; **Supplementary Figure 5**).

##### 3.2.1.4 No differences in regulatory T cells (Tregs), T follicular helper cells (Tfh), T follicular regulatory (Tfr) cells or cytokine producing subsets (Th1, Th2, Th17) in IOPD patients treated with ERT+MTX

A comparison of other immune parameters in CD4^+^ cells was carried out, including frequencies of Th1, Th2, and Th17 cells, regulatory T cells (Treg; CD4^+^CD25^+^CD127^-^), T-follicular helper cells (Tfh; CD4^+^CXCR5^+^CD45RA^-^), or T-follicular regulatory cells (Tfr; CD4^+^CXCR5^+^CD45RA^-^CD127^-^CD25^+^), but did not reveal a significant difference between IOPD patients who were treated with ERT+MTX and those that were untreated (**Table 2**; **Supplementary Table 3**; **Supplementary Figure 7**).

##### 3.2.1.5 Lower TIGIT^+^ CD8^+^ T cells in ERT+MTX treated IOPD patients

We also evaluated the expression of the inhibitory co-receptors TIGIT (T cell Immunoreceptor with Ig and ITIM domains) and PD-1 (Programmed cell death protein 1) on CD4^+^ and CD8^+^ T cells. In comparison to the ERT+MTX untreated group, we observed significantly fewer TIGIT^+^ CD8^+^ T cells in the ERT+MTX treated group (0.49% vs. 2.25% of CD8^+^ T cells; P = 0.011) (**Figure 4D**). However, there was no difference between treated and untreated groups in PD-1^+^ cells (**Table 2**).

We carried out phenotypic analysis on TIGIT^+^ cells. While the TIGIT^+^ CD8^+^ population was predominantly naïve both before (67.1 ± 6.4%) and after (59.0 ± 18%) ERT+MTX treatment, there was a relatively lower frequency of central memory cells in the ERT+MTX-treated group compared to the untreated group (1.8% vs 12.7%; P = 0.007). Among untreated TIGIT^+^ CD4^+^ cells, naïve (51.1 ± 5.9%) and central memory (39.6 ± 4.8%) cells predominate, but were not statistically significant different from the treated group. Since TIGIT is associated with Tregs and TFH cells, we determined that Tregs represented 31.4 ± 5.4% and TFH represented 9.7 ± 1.8% of TIGIT^+^ CD4^+^ cells in the untreated group, and that these frequencies were not significantly different in the treated group (Tregs 30.3 ± 11.6% and THF 12.4 ± 5.6% post-treatment). We examined co-expression PD-1 on TIGIT^+^ cells and found that on average ~1-15% of CD4^+^TIGIT^+^ cells and 1-12% of CD8^+^TIGIT^+^ cells co-expressed PD-1 in untreated patients, and that there was no difference in TIGIT^+^PD-1^+^ cells between untreated or treated groups (**Supplementary Figure 8**).

##### 3.2.1.6 Pompe disease activity increases with age in children with IOPD prior to ERT+MTX treatment

Urinary Glc_4_ (glucosyl tetrasaccharide) is a glycogen-derived oligosaccharide that accumulates due to impaired lysosomal degradation of glycogen in Pompe disease. Elevated Glc_4_ reflects glycogen buildup and is used as a non-invasive biomarker for disease burden (20). Its levels correlate with disease activity and are commonly used as a pharmacodynamic marker to monitor response to enzyme replacement therapy (ERT) (18, 20). We aimed to evaluate whether ERT and TLD-MTX treatment would have an impact on the Glc_4_ level, as a reflection of disease activity, in IOPD patients.

**Figure 5:**
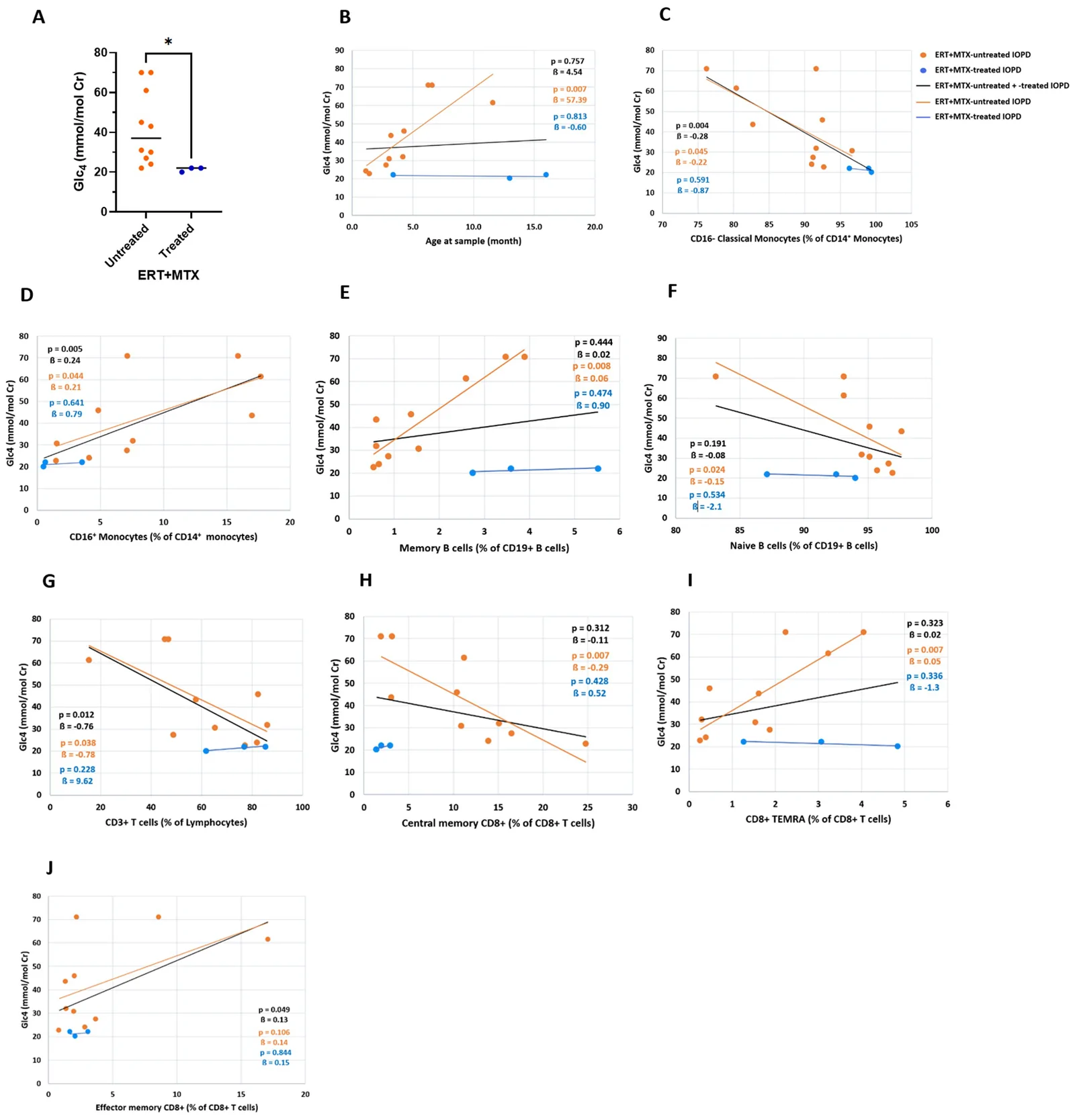
Correlation between Glc_4_ levels and immune cell subsets in IOPD patients undergoing ERT+MTX therapy. **(A)** Comparison of Glc_4_ levels in IOPD patients at baseline and post-ERT+MTX. Statistical comparisons were performed using the Mann-Whitney test. *P < 0.05, **(B–J)** Correlation analysis (using Simple Linear Regression) between Glc_4_ levels (mmol/mol Cr) and various immune cell subsets was performed for ERT+MTX- untreated and treated IOPD patients. Panels show the relationships for each subset: **(B)** age at sample (month), **(C)** CD16^-^ classical monocytes, **(D)** CD16^+^ monocytes, **(E)** Memory B cells, **(F)** Naive B cells, **(G)** CD3^+^ T cells, **(H)** Central memory CD8^+^ T cells **(I)** TEMRA CD8^+^ T cells, and **(J)** Effector memory CD8^+^ T cells. Pre-ERT+MTX samples are represented in orange, and post-ERT+MTX samples in blue. Regression lines for each group are added for visual aid and color-coded as follows: black for combined ERT+MTX-untreated and -treated IOPD, orange for ERT+MTX-untreated IOPD only, and blue for ERT+MTX-treated IOPD only.

There were significantly lower mean Glc_4_ levels in IOPD patients after treatment, compared to pre-treatment levels (**Figure 5A**; P = 0.017). Within the narrow age range in IOPD patients (1–17 months) simple linear regression demonstrated that Glc_4_ levels increased significantly with age in the untreated group (**Figure 5B**; P = 0.007), demonstrating accumulation of glycogen over the first year of life. **Figure 5B** demonstrates that inclusion of the post-treatment group (which was older on average than the pre-treatment group) in regression analysis results in loss of this correlation, clearly due to the lower Glc_4_ activity in the treated group (pre-ERT+MTX: β= 57.394, 95% CI [20.69 to 94.09], P = 0.007; post-ERT+MTX: β= -0.609, 95% CI [-26.21 to -24.99], P = 0.813; pre+post ERT+MTX: β = 4.541, 95% CI [-27.01 to 36.10], P = 0.757).

##### 3.2.1.7 Pompe disease activity is associated with a pro-inflammatory and activated immune profile in IOPD

To identify immunological correlates of disease in IOPD, we explored potential associations between Glc_4_ levels and immune markers within the IOPD cohort. We demonstrated a strong correlation between age and disease activity in untreated IOPD patients that was negated by ERT+MTX treatment, suggesting that age was a surrogate for disease activity in these children (**Supplementary Tables 5**, **6**). To avoid masking correlations between immune parameters and disease activity, we carried out further analyses using simple linear regression between Glc_4_ and immune parameters. While age was not accounted for in the primary analysis, multiple regression accounting for age was also carried out, results are presented in **Supplementary Table 7**, and correlations that were consistent between simple and multiple regression are indicated below by an asterisk (*). Given the clear and profound effect of ERT+MTX treatment on disease activity (i.e., Glc_4_ levels), we carried out separate analyses in the untreated (pre-MTX+ERT) and treated (post-MTX+ERT) groups. For comparison, we also carried out regression analysis on the entire IOPD cohort that includes both pre- and post-treatment samples (pre+post ERT+MTX).

In untreated IOPD patients, we found a significant negative correlation between Glc_4_ levels and the frequency of classical CD14^+^CD16^−^ monocytes and a positive correlation with the frequency of CD16^+^ (non-classical and intermediate) monocytes (β= -0.225, 95% CI [-0.442 to -0.007], P = 0.045 for classical monocytes, β= -0.214ß, 95% CI [0.008 to 0.420], P = 0.044 for CD16^+^). These correlations were also significant in regression analysis of the whole treated and untreated cohort (i.e., pre+post ERT+MTX; β= -0.281, 95% CI [-0.451 to -0.112], P = 0.004 for classical monocytes*, β= 0.242, 95% CI [0.091 to 0.393], P = 0.005 for CD16^+^ monocytes*) (**Figures 5C, D**; **Supplementary Table 6**). No significant correlation was found in the treated samples alone; however, interpretation of results for the post-treatment cohort should consider the small number of patients (n=3).

We also found that, in ERT+MTX untreated IOPD patients, Glc_4_ levels were positively correlated with the frequency of mature memory B cells (β= 0.062, 95% CI [0.039 to 0.084], P = 0.008*) (**Figure 5E**; **Supplementary Table 6**) but negatively correlated with the frequencies of naïve B cells (β= -0.155, 95% CI [0.283 to 0.026], P = 0.024) and plasmablasts (β= -0.305, 95% CI [-0.568 to -0.042], P = 0.028) (**Figure 5F**; **Supplementary Table 6**). There was no significant correlation with these B cell parameters in treated patients or when analysis was carried out on the whole cohort of treated + untreated patients.

Disease activity (Glc_4_) correlated with a lower percentage of T cells in untreated patients (β= -0.787, 95% CI [-1.520 to -0.054], P = 0.038) and in the whole treated + untreated cohort (β= -0.765, 95% CI [-1.308 to -0.222], P = 0.010). Further analysis of T cell subsets showed that Glc_4_ levels were negatively associated with central memory CD8^+^ T cells (β= -0.296, 95% CI [-0.489 to -0.104], P = 0.007*) and positively associated with TEMRA CD8^+^ T cells (β= 0.055, 95% CI [0.020 to 0.090], P = 0.007) in untreated patients. In the whole cohort, Glc_4_ levels were positively associated with effector memory CD8^+^ T cells (β= 0.133, 95% CI [0.000 to 0.265], P = 0.049) (**Figures 5G–J**; **Supplementary Table 6**).

#### 3.2.2 Immune phenotyping in LOPD cohort

Samples were taken from patients in LOPD cohort before (i.e., pre-ERT+MTX) and ~6 months after (i.e. post-ERT+MTX) initiation of treatment with ERT+MTX. Immune profiling was carried out using the same panels described for IOPD patient samples.

##### 3.2.2.1 Decreased B cells after treatment with ERT+MTX in LOPD patients

**Figure 6:**
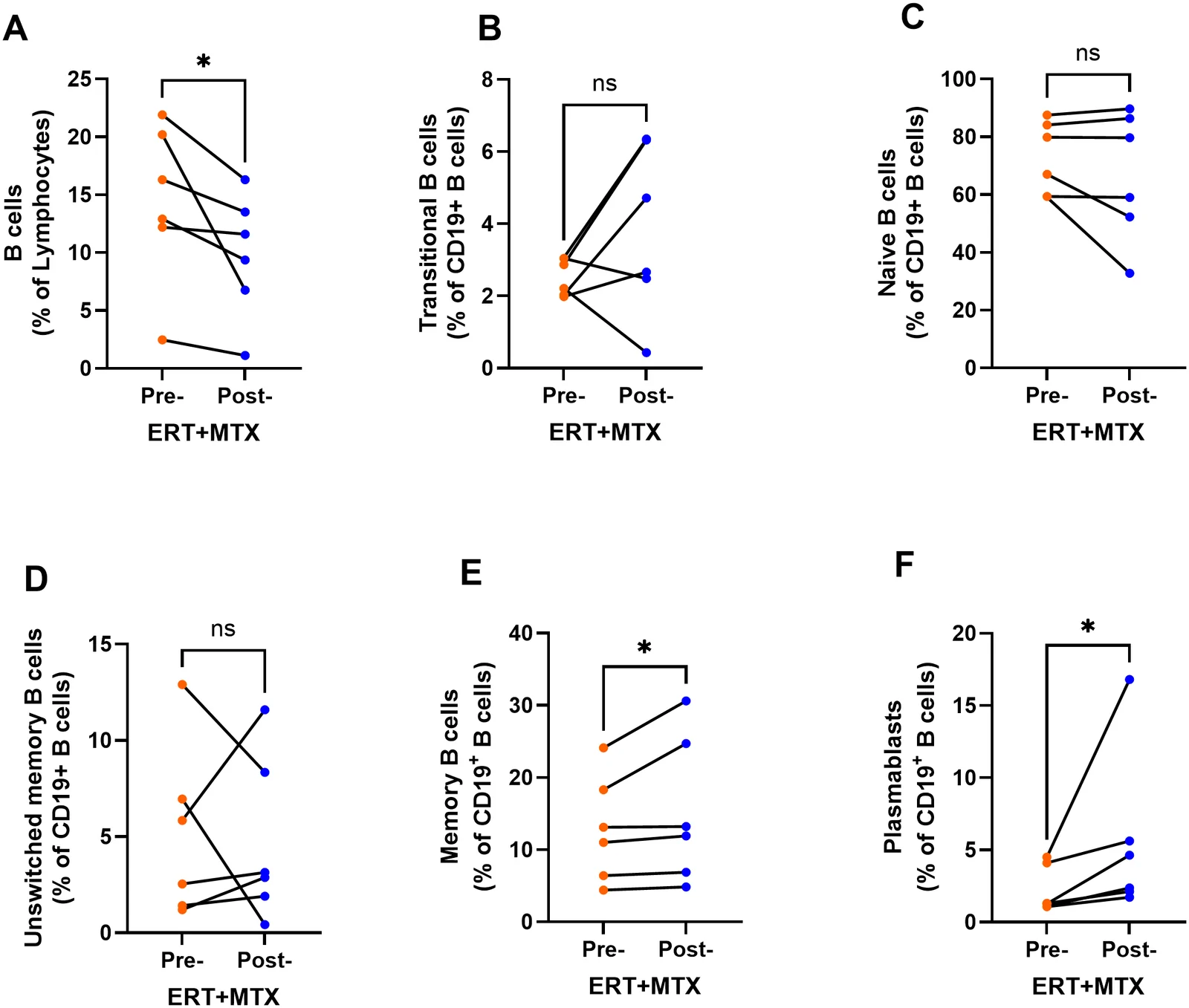
Increase in memory B cells and immature plasma cells in the post-ERT+MTX group of LOPD patients. Comparison of antibody-secreting B cell subpopulations (as percent of CD19^+^ B cells) in LOPD patients at pre- and post-ERT+MTX treatment, including: **(A)** CD19^+^ B cells **(B)** transitional B cells (CD19^+^CD20^+^CD38hi CD24hi), **(C)** naïve B cells (CD19^+^IgD^+^CD27^-^), **(D)** unswitched memory B cells (CD19^+^IgD^+^CD27^+^), **(E)** memory B cells (CD19^+^IgD^-^CD27^+^), and **(F)** plasmablasts (CD19^+^CD27^+^IgD^-^CD20^-^CD38^++^). Pre-ERT+MTX samples are represented in orange, and post-ERT+MTX samples in blue. Statistical comparisons were performed using the Wilcoxon signed-rank test. *P < 0.05, “ns” indicates a non-significant difference.

We evaluated potential differences in circulating immune subsets between pre- and post-ERT+MTX treatment in LOPD patients. Specifically, we wanted to understand whether treatment with ERT+MTX was associated with changes in the relative abundance of B cell subsets within the B cell compartment. To do so, we carried out gating to identify naïve, transitional, unswitched memory, and switched memory B cells and plasmablasts (early antibody-secreting cells CD19^+^CD27^+^IgD^-^CD20^-^CD38^++^) and calculated their relative frequency using the total B cell gate as the denominator representing the whole B cell compartment (**Supplementary Figure 3B** for gating, **Table 2** for mean/SD of pre- and post-treatment groups). This approach allows for detection of relative changes between B cell subtypes which can suggest activation or maturation of B cells, but given the single and static nature of the data, caution must be taken when inferring a dynamic processes. We detected a modest but statistically significant decrease in the overall frequency of CD19^+^ B cells compared to untreated patients (14.32% vs. 9.77% of lymphocytes; P = 0.032) (**Figure 6A**) relative to its parent gate, the total lymphocyte gate. This decrease may reflect either a true decrease in B cells or relative increase in T cells within this gate (65.36 vs 71.71%) though the difference in T cells failed reach significance. Of important note, this relative decrease in %CD19^+^ B cells cannot be used to infer a decrease in absolute B cell count in the absence of direct measurement. Similar to the changes we detected in IOPD patients, the percent of mature memory B cells relative to other B cell subsets were increased in LOPD patients after ERT+MTX treatment (15.32% vs. 12.88% of B cells, P value=0.046). In contrast to IOPD, however, LOPD patients also had relatively increased circulating plasmablasts after treatment (5.38% vs. 2.22% of IgD^-^ B cells, P = 0.046) (**Figures 6E, F**).

##### 3.2.2.2 No differences in Tregs, TFh, TFr cells or cytokine producing subsets (Th1, Th2, Th17) in LOPD patients treated with ERT+MTX

A comparison of other immune parameters including frequencies of Th1, Th2, Th17 Treg, Tfh, and Tfr cells, did not reveal a significant difference between LOPD patients before and after treatment within the time interval of the samples we obtained (**Table 2**; **Supplementary Figure 7**).

##### 3.2.2.3 Increased TIGIT^+^ CD4^+^ T cells after treatment with ERT+MTX in LOPD patients

**Figure 7:**
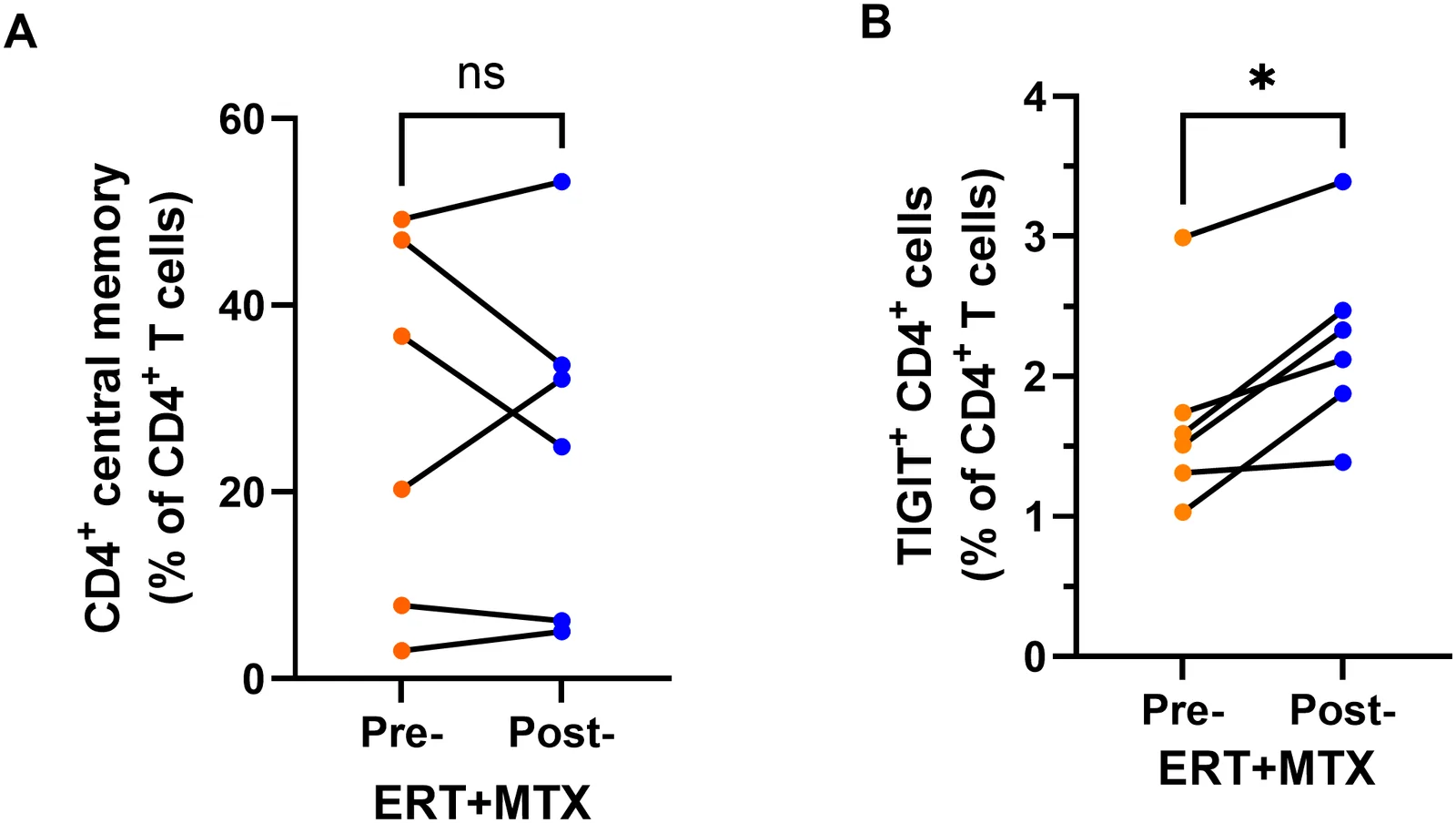
Enhanced TIGIT expression on CD4^+^ T cells in post-ERT+MTX LOPD group. Comparison of CD4^+^ T cells, including: **(A)** CD4^+^ central memory T cells, **(B)** TIGIT^+^ CD4^+^ T cells. Pre-ERT+MTX samples are represented in orange, and post-ERT+MTX samples in blue. Statistical comparisons were performed using the Wilcoxon signed-rank test. *P < 0.05, “ns” indicates a non-significant difference.

LOPD patients treated with ERT+MTX exhibited a slight but statistically significant increase in CD4^+^ T cells expressing the inhibitory co-receptor TIGIT after treatment, compared to pre-ERT+MTX levels (2.26% vs. 1.69% of CD4^+^ T cells; P = 0.028) (**Figure 7**).

We carried out phenotypic analysis on TIGIT^+^ cells in LOPD patients. Maturational stage analysis of the TIGIT^+^ CD4 population revelated that there were wide differences between members of the cohort, with the general distribution in the untreated group being 24.1 ± 11.1% naïve, 44.9 ± 8.8% central memory, 24.8 ± 5.5% effector memory, and 6.1 ± 3.1% TEMRA, and there was no detectable significant difference between groups post-treatment. Since TIGIT is expressed on a subset of Tregs and Tfh cells, we determined that Tregs represented 29.5 ± 4.2% and Tfh represented 7.5 ± 3.2% of TIGIT^+^ CD4^+^ cells in the untreated group, and that these frequencies were not significantly different in the treated group (Tregs 27.2 ± 4.2% and THF 7.1 ± 3.8% post-treatment; see **Supplementary Figure 6**). We examined co-expression PD-1 on TIGIT^+^ cells and found approximately that ~5-15% of CD4^+^TIGIT^+^ cells and 5-24% of CD8^+^TIGIT^+^ cells co-expressed PD-1 in untreated patients, and that while cells percents changed in individual patient, there was no significant difference in TIGIT^+^PD-1^+^ cells in patients pre- and post-treatment as a group (**Supplementary Figure 8**).

##### 3.2.2.4 Correlation of Glc_4_ and immune profile in LOPD cohort

**Figure 8:**
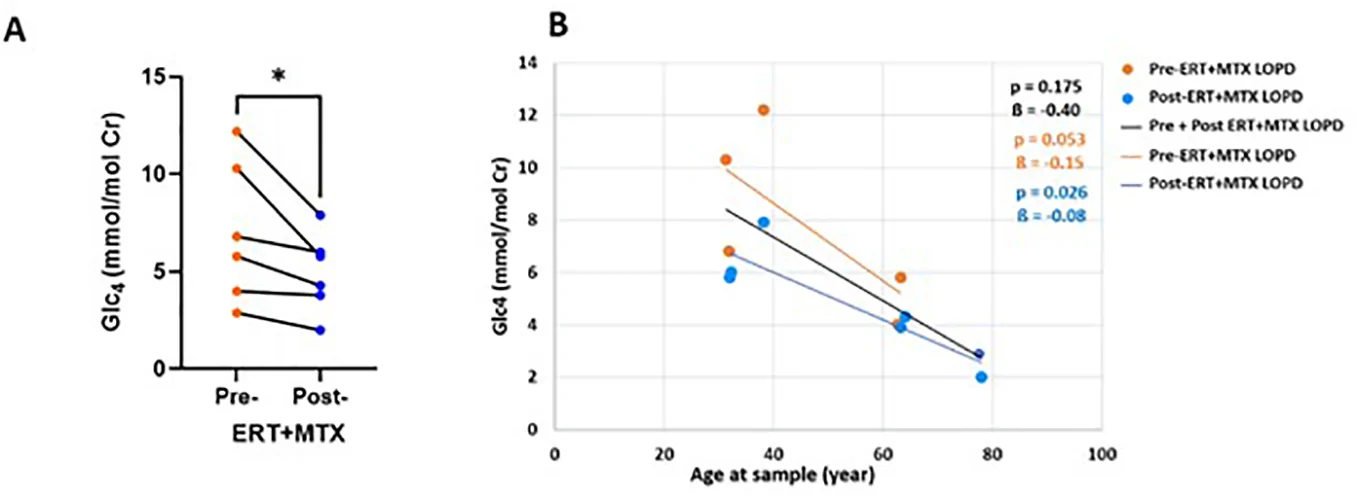
Correlation between Glc_4_ levels and age in LOPD patients undergoing ERT+MTX therapy. **(A)** Comparison of Glc_4_ levels across LOPD patients. Statistical comparisons were performed using the Wilcoxon test. *P < 0.05, **(B)** Linear regression of Glc_4_ vs. age. Regression lines for each group are added for visual aid and color-coded as follows: black for combined Pre + Post-ERT+MTX LOPD, orange for Pre-ERT+MTX LOPD only, and blue for Post-ERT+MTX LOPD only.

There was a significant decreased Glc_4_ in LOPD patients after treatment with ERT+MTX (P = 0.035), reflecting clearance of glycogen and decreased disease activity (**Figure 8A**). Of note, Glc_4_ levels in the LOPD groups were significantly lower than in the IOPD group (6.01 vs. 37.5, P = 0.001), with the highest level in the LOPD patients measuring lower than the lowest measurements in IOPD patients. This finding is consistent with the different clinical severity of both glycogen accumulation and disease severity in IOPD vs. LOPD, as we have shown previously (21). To understand whether there was a unique association between disease activity and immunological parameters in LOPD, we carried out correlation analysis of patient samples before (pre-ERT+MTX) and after (post-ERT+MTX) initiation of ERT+MTX therapy.

##### 3.2.2.5 Glc_4_ does not correlate significantly with most immune parameters in LOPD patients in age-adjusted analysis

Given that there was a wide range of ages in the LOPD cohort at the time of initiation of therapy, we examined the relationship between age and baseline Glc_4_ (**Figure 8B**), demonstrating that Glc_4_ serum measurements were not significantly associated with age in untreated patients (β= -0.150, 95% CI [-2.79 to 0.049], P = 0.053), but that a subtle yet significant negative association was noted after initiation of therapy (β= -0.089, 95% CI [-0.161 to -0.017], P = 0.026). Given the inability to exclude age as a potential confounding factor in the LOPD cohort, we compared both simple logistic regression and multiple logistic regression, adjusting for age (**Supplementary Tables 8**, **9**). While multiple immune parameters appeared on first analysis unadjusted (simple) regression to be correlated with Glc_4_ (e.g., monocyte and lymphocyte subsets), many of these factors were also correlated independently with age and did not prove to be significant upon multiple regression analysis accounting for age. The only parameter that was found to correlate with Glc_4_ levels on multiple regression was the percentage of CD4^+^CD45RA^-^CXCR5^+^CD25^+^CD127^-^ T follicular regulatory (TFr) cells, which were negatively correlated with Glc_4_ levels (β= -0.602, 95% CI [-0.1671 to -0.034], P-value 0.043) in the untreated LOPD cohort only. After treatment, there was no correlation between Tfr and Gcl_4_ (**Supplementary Table 9**).

##### 3.2.2.6 High anti-rhGAA antibody titers demonstrate a breach in tolerance in three patients.

**Figure 9:**
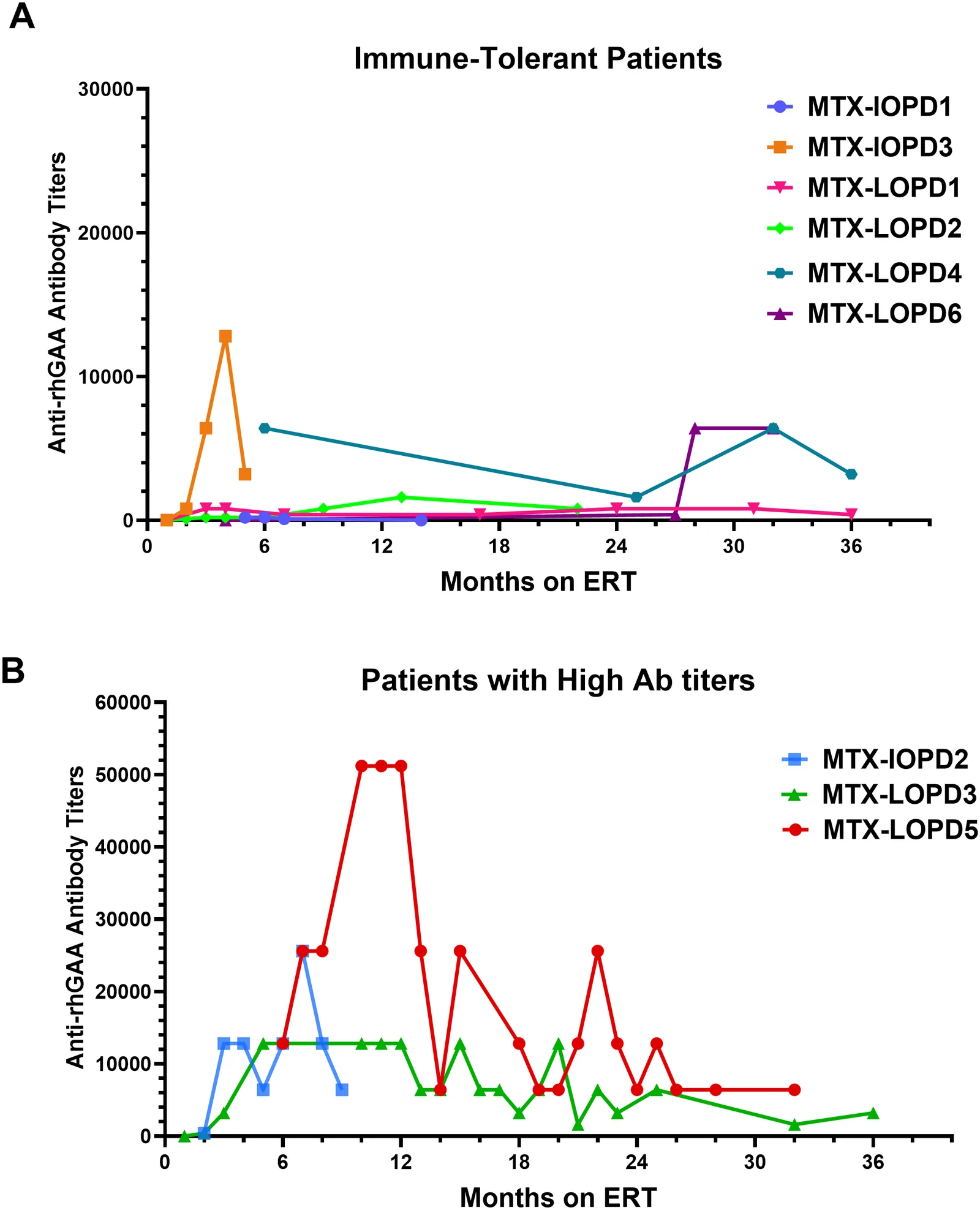
Anti-hGAA Antibody Titers in IOPD and LOPD Patients Receiving TLD-MTX. **(A)** Immune-tolerant patients who maintained low anti-hGAA titers. **(B)** Patients who developed high antibody titers. Each line represents an individual patient’s anti-hGAA titer measured at the indicated months on ERT.

Detection of high antibody titers >12,800 during treatment with ERT represents a break in the tolerance that was previously established during immune tolerance induction with TLD-MTX. Antibody titers were evaluated in all IOPD patients to assess immune responses to treatment. Among them, only patient MTX-IOPD2 exhibited developing antibody titers of 12,800 at months 3 and 8, which peaked at 25,600 at month 6 before declining to 6,400 by month 9 (**Figure 9**).

In the LOPD cohort, TLD-MTX induced lasting immune tolerance in 4 out of 6 patients (except patients MTX-LOPD3 and MTX-LOPD5), defined as the absence anti-rhGAA antibody titers. MTX-LOPD3 exhibited antibody titers ranging from 12,800 to 51,200 between months 5 and 20, while patient MTX-LOPD5 showed a similar pattern, with titers fluctuating between 12,800 and 51,200 from months 6 to 25 before declining to 6,400 by month 26 (**Figure 9**). All other patients receiving methotrexate maintained low antibody titers (<12,800) throughout the follow-up period (**Supplementary Table 2**).

### 3.3 Immune phenotyping in patients with high antibody titers

**Figure 10:**
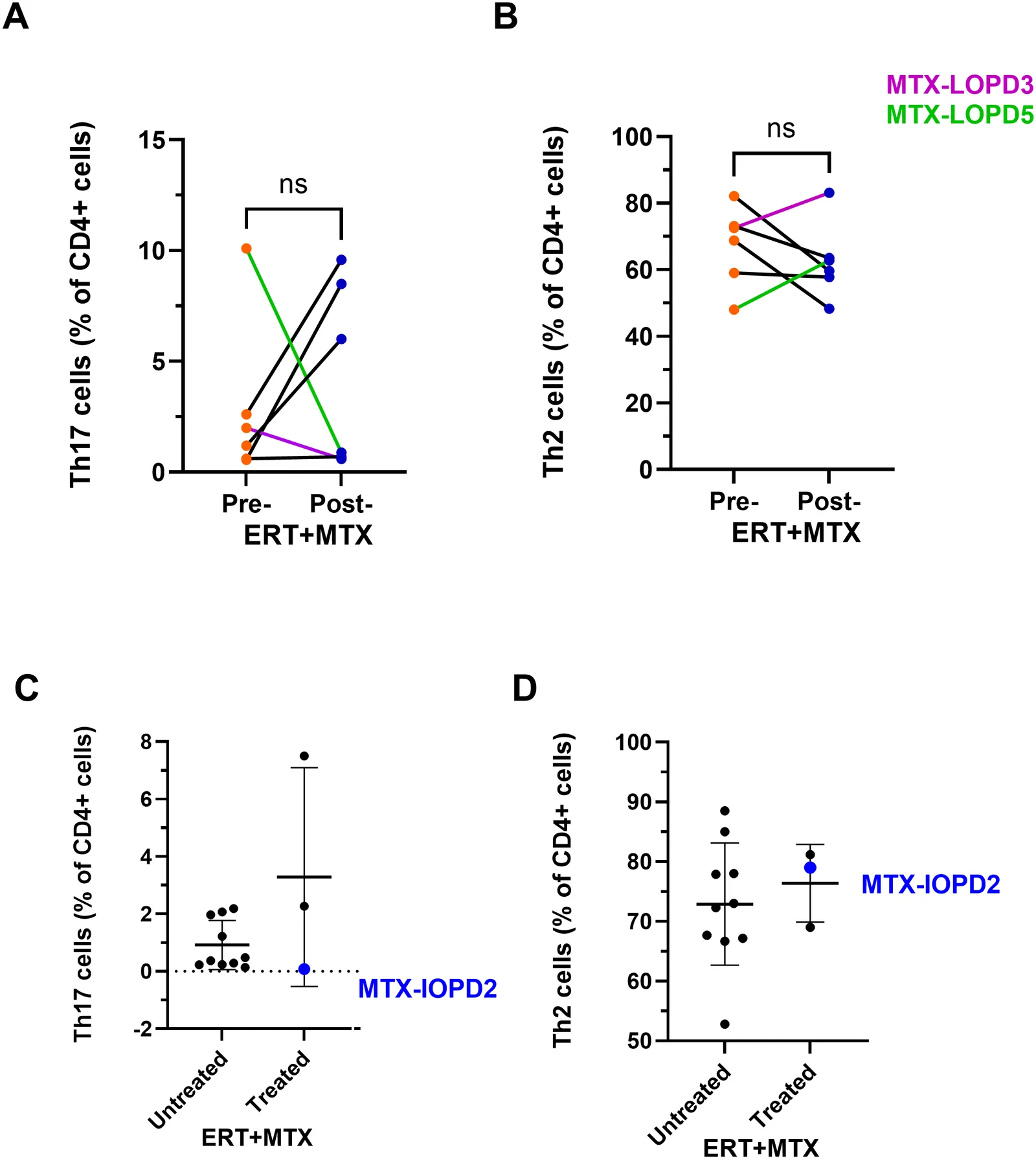
Common immune phenotypes in patients with HSAT. **(A, B)** Comparison of T helper 2 (Th2; CXCR3^-^CCR6^-^) and T helper 17 (Th17; CXCR3^-^CCR6^+^) cells between pre- and post-ERT+MTX samples in LOPD patients who developed high antibody titers. Two LOPD patients (MTX-LOPD3 and MTX-LOPD5) showed increased circulating Th2 cells and decreased Th17 cells after treatment. **(C, D)** Comparison of Th2 and Th17 cells between ERT+MTX-untreated and -treated samples in IOPD patients who developed high antibody titers. One IOPD patient (MTX-IOPD2) showed increased circulating Th2 cells and decreased Th17 cells after treatment. Bars represent mean values for each group.

We sought to understand whether there were immune phenotypic characteristics of patients who developed high antibody titers that could suggests clues regarding the immunological origins of their broken tolerance. Previous studies in GAA-deficient mice treated with ERT using rhGAA demonstrated that anti-GAA IgG antibody responses are primarily driven by immunodominant epitopes recognized by CD4^+^ T helper type-2 (Th2) cells (22). We extended and confirmed this finding to human cohorts, demonstrating that blood samples from IOPD patients who developed HSAT exhibit increased Th2 cells and decreased Th17 cells compared to those who did not break tolerance (14). Based on this evidence, we quantified T helper (Th) subtypes in patients in the current study utilizing well-established chemokine receptor expression patterns to identify distinct populations of CD4^+^ effector cells enriched for Th1 (CXCR3^+^CCR6^-^), Th2 (CXCR3^-^CCR6^-^), and Th17 (CXCR3^-^CCR6^+^) cells. Unlike other patients in the LOPD cohort who did not develop high titers, we found that Th17 cells were decreased in patients with high titers after treatment, compared with baseline samples (0.60% vs. 1.99% of CD4^+^ cells in MTX-LOPD3 and 0.88% vs. 10.1% of CD4^+^ cells in MTX-LOPD5) (**Figure 10A**). In contrast, Th2 cells in these patients were increased after treatment compared with baseline samples (72.5% vs. 83.1% of CD4^+^ cells in MTX-LOPD3 and 48.0% vs. 62.8% of CD4^+^ cells in MTX-LOPD5) (**Figure 10B**). In MTX-IOPD2, the only IOPD patient to develop high titers, a matched baseline sample was not available; however, the frequency of Th17 cells in this ERT+MTX-treated patient’s sample was lower than the mean frequency observed in unmatched baseline samples (0.92% vs. 0.08%) (**Figure 10C**).

## 4 Discussion

Multiple immunomodulation strategies have been used to counteract the anti-rhGAA IgG antibody response, with varying degrees of success (9, 23). This study evaluated an ITI protocol using a TLD-MTX that was started alongside ERT in treatment-naïve CRIM-positive IOPD and LOPD patients (10). While several patients on this regimen maintained low antibody titers, one IOPD patient (MTX-IOPD2) developed HSAT antibodies by three months, while two LOPD patients (MTX-LOPD-3 and MTX-LOPD5) reached HSAT levels at six months.

MTX, an inhibitor of dihydrofolate reductase, disrupts purine nucleotide and thymidylate synthesis that is required for DNA replication and repair, thereby inhibiting proliferation and causing death of rapidly-dividing cells like activated lymphocytes (24). Previous studies have demonstrated that low-dose MTX regimens can effectively reduce anti-drug antibody responses across various protein therapies in normal and disease contexts (10, 25). There are several mechanisms by which MTX has been shown to affect B and T lymphocytes in mouse or human studies, including increased extracellular adenosine, cell cycle arrest, apoptosis, and reduced levels of adhesion molecules and nitric oxide (26, 27). Although MTX has shown efficacy in Pompe disease, little is known about the immunological state of Pompe patients at baseline, and the exact mechanism of MTX-induced immune tolerance induction remains unclear. The primary goal of this study was to conduct a comprehensive immune profiling of cell subsets to identify unique immunological features at baseline and following TLD-MTX administration in an ERT-naïve setting in patients with IOPD and LOPD. This approach aims to enhance our understanding of the cellular subsets that are affected by MTX therapy, but also to potentially gain insight into immunological features associated with disease progression.

A potential caveat to consider in the interpretation of this study is that we do not have the ability to compare absolute cell counts between treated and untreated patients. While both relative frequency and absolute counts are used in flow cytometry studies (28), relative percentages of cell subsets are typically employed to detect shifts in the relative frequency of populations within a cell compartment, including maturation and activation subsets. An important exception is in cases of lymphocyte depletion or immune reconstitution where biologically important deficits of immune subsets can be masked, for example, by an apparent preservation of the percentage of memory T or B cells in the setting of profoundly depleted absolute counts of T or B cells (28). Methotrexate can be associated with cytopenias, including B cell lymphopenia, during prolonged use for immunosuppression. Even low-dose methotrexate administered weekly to patients with lupus erythematosus for greater than 10 weeks was reported to induce a slight decrease in B cell counts, but this was not seen in patients treated for less than 10 weeks (29). However, another study of patients with Juvenile Idiopathic Arthritis who received low-dose methotrexate weekly for at least 6 months did not demonstrate a decrease of absolute B cell counts compared to untreated controls (30). In our study, transient low-dose methotrexate is given as a single treatment during immune tolerance induction (3 days of daily methotrexate given every week or every other week, based on ERT frequency, for 3 ERT doses, i.e., 9 doses total) 6 months prior to sample acquisition from the treated IOPD group and the post-treatment LOPD patients, and we therefore do not expect any methotrexate-related cytopenias to influence results of these studies.

### 4.1 Therapeutic ERT+MTX in IOPD: key findings and ongoing debates

There is a great deal of phenotypic and functional diversity in monocyte function, even within the generally accepted phenotypic divisions based on the surface markers CD14 and CD16 (31–33). In this study, IOPD patients treated with ERT+MTX exhibited reduced CD16^+^ monocytes and increased classical CD14^+^CD16^-^ monocytes compared to pre-treatment patients. Classical monocytes, the most abundant subset of circulating monocytes, represent a nascent population that has recently emerged from the bone marrow and which subsequently undergoes sequential maturation first into intermediate monocytes as they acquire CD16^+^ expression and ultimately into CD16^+^CD14^-^ non-classical monocytes (34). Classical monocytes are generally thought to be less inflammatory than CD16^+^ subsets, and while they play an important role in initiating inflammatory responses to bacterial infections and other sources of tissue inflammation, they also are thought to play a role in resolution of inflammatory responses and tissue repair (32, 33). Intermediate and non-classical monocytes, generally considered to be more pro-inflammatory subsets, play a key role in innate inflammatory responses and also promote T cell proliferation, with the SLAN^+^ non-classical subset particularly involved in stimulating CD4^+^ T cell responses (19, 33). Multiple studies have shown that increased levels of circulating CD16^+^ non-classical and intermediate monocytes are associated with worse outcomes in inflammatory disease, including autoimmune diseases (35, 36). Reports in kidney transplant patients indicate that elevated levels of CD16^+^ monocytes persist even under immunosuppressive therapy, potentially signifying an allograft-induced inflammatory response (35). The prominence of CD16^+^ monocyte subsets across various inflammatory diseases further underscores their involvement in chronic inflammation (35–37). In our IOPD cohort, the reduced CD16^+^ monocyte percentage in the ERT+MTX treated group could, therefore, reflect a therapeutic effect of ERT+MTX in reducing inflammation.

An important question arises from these observations whether any observed effects of immune phenotype after ERT+MTX treatment is due to the direct immunosuppressive effects of MTX vs. the results of reducing glycogen accumulation as measured by urine Glc_4_ via ERT. This leads us further to ask whether there is a direct inflammatory effect of glycogen buildup in Pompe disease. GAA deficiency in Pompe disease leads to the accumulation of glycogen within lysosomes. Excess glycogen that is eventually released into the circulation from damaged tissue is partially degraded into a stable limit dextrin glucose tetrasaccharide, or Glc_4_, which is efficiently excreted into the urine (38). Monitoring urinary Glc_4_ levels provides a valuable tool for evaluating disease progression and the effectiveness of ERT (39). Since the levels of glycogen buildup (reflected as circulating Glc_4_) are greatly exacerbated in IOPD compared to LOPD, any direct immune effects of accumulated tissue glycogen may be more pronounced in these IOPD patients, as might any salutary effects of treating the disease with ERT. Given that ERT and MTX are administered simultaneously in the regimen reported in this study, it is impossible to say with certainty whether the effect of treatment is due to one or the other. We may, however, look to other immunophenotyping studies using TLD-MTX only (i.e. without ERT) to infer the source of effects. In a recent study of people living with HIV, immune profiling was carried out on subjects enrolled in a placebo-controlled trial of TLD-MTX to treat inflammation-associated comorbidities of HIV (e.g., cardiovascular disease) (40). This study demonstrated that TLD-MTX treatment decreased proliferating T cells, but did not alter the percentage of monocyte subsets or reduce levels of plasma inflammatory cytokines. This may suggest that the shift toward a less pro-inflammatory monocyte population that we observed in IOPD patients reflects the impact of ERT on glycogen-induced inflammation. Further supporting the relationship between Pompe disease activity and inflammation, we demonstrated a strong correlation between higher Glc_4_ and increased CD16^+^ monocytes (with decreased classical monocytes) that was persistent even in multiple regression accounting for age. Together, these results suggest that ERT not only treats the direct consequences of glycogen accumulation in tissues (e.g., muscle weakness) but also modifies the inflammatory response triggered by glycogen buildup. While this remains speculative, we hypothesize that glycogen accumulation itself may contribute to baseline immune activation in Pompe disease. This hypothesis will be directly tested in ongoing studies comparing pre-treatment immune profiles in early childhood IOPD and LOPD patients with non-affected, matched controls.

We observed higher mature memory B cells and lower plasmablasts in the ERT+MTX treated IOPD group compared to the pre-treatment group. Plasmablasts are proliferative antibody secreting cells (ASC) that emerge in response to infection or inflammatory stimuli, represent an early-intermediate form of ASC, and typically surge in the setting of acute immune responses (41, 42). Mature memory B cells, on the other hand, have progressed through isotype switching and maturation, typically in a germinal center or secondary lymphoid tissue. The lower percent of plasmablasts in the ERT-MXT treated group, therefore, could reflect a therapeutic effect due to the direct anti-proliferative effect of MTX in this proliferative population and/or a relative dampening of the putative inflammation associated with glycogen accumulation. The increased frequency of mature memory B cells after treatment may suggest a shift from an inflammatory immune response to a more regulated, memory-focused immune state (43). It may also suggest that a healthier tissue mileu, in the absence of excess glycogen buildup, is more supportive of functional germinal center formation. This balance, with fewer actively secreting plasmablasts and a greater pool of mature memory cells, suggests that the treatment may aid in achieving immune homeostasis (43, 44).

While all IOPD patients in this study are under 17 months of age, the mean age of the post-treatment group is significantly older than the pre-treatment group. A consistent challenge of these analyses is to discern whether observed effects are attributable to age, disease, or treatment. This question is especially relevant in the IOPD group because many normal changes occur in the immune system during the first years of life (45). We attempted to partly address this challenge by incorporating age in our analysis, including linear regression with and without age adjustment. In correlation analyses of the pre-treatment group, high Glc_4_ correlated with low plasmablasts, low naïve B cells, and high mature memory B cells (**Supplementary Table 5**). Increased memory B cells and decreased plasmablasts cells may be expected as a part of normal maturation in the first year of life and were found to correlate with age in the pre-treatment group (**Supplementary Table 5**). However, the positive correlation between Glc_4_ and memory B cells (but not plasmablasts) remained significant in the pre-treatment group after age adjustment in multiple regression (**Supplementary Tables 5**-**7**). A simple regression of mature B cell % vs. age including the whole cohort was not significant, also suggesting that the B cell correlation with Glc4 is not attributable to age (**Supplementary Table 5**). Further comparison of IOPD and control patients along the spectrum of development, ideally in conjunction with mechanistic studies, are needed to discern whether the observed changes are attributable to normal immune development or to Pompe Disease.

Regarding T cell subsets, our finding indicated a decrease in central memory CD4^+^ and CD8^+^ T cells in the ERT+MTX treated IOPD group. Central memory T cells are crucial for maintaining a reservoir of antigen-experienced T cells that can rapidly respond to re-exposure (46, 47). They are generated following activation of naïve T cells during an active immune response. A reduction in this subset after ERT+MTX treatment could reflect the effects of MTX on proliferating cells. While central memory cells have high proliferative potential, they themselves are minimally proliferative. However, exposure to MTX could potentially curtail the generation of new central memory cells, and therefore the post-treatment decrease in central memory cells could reflect such an interrupted immune response. The extent to which we may expect such an effect after a short course (nine doses) of low dose MTX six months prior to sample analysis is unknown. Examination of correlations between Glc_4_ and T cell maturational subsets may suggest a more nuanced scenario, however.

In pre-treatment patients, Glc_4_ is positively correlated with higher effector and terminal-effector memory (i.e., TEMRA) CD8^+^, and negatively correlated with central memory CD8^+^ cells in simple regression analysis. This could suggest that glycogen accumulation in IOPD patients is associated with T cell activation, resulting in effector-memory and terminal effector differentiation, possibly at the expense of acquiring a healthy central memory population. This type of activation can be seen in cases of bystander immune activation leading to immune exhaustion. This could also be supported by the relatively higher expression of TIGIT on CD8^+^ cells that we detected in pre-treatment patients, compared to post-treatment patients (48, 49). TIGIT is an inhibitory receptor that can be expressed by effector T cells, natural killer (NK) cells, T regulatory cells (Treg), and T follicular helper (Tfh) cells and typically functions to dampen T cell activity and maintain immune tolerance, particularly under conditions of chronic immune stimulation (50, 51). In addition to finding lower %TIGIT^+^ cells in the treated group, we found that there was a relative decrease in the % of TIGIT cells that were central memory phenotype. Lower levels of TIGIT^+^ CD8^+^ T cells may indicate a less activated or less exhausted immune profile, as TIGIT is often upregulated in states of persistent inflammation or immune exhaustion (52). The reduction in TIGIT-expressing cells and decreased central memory T cells suggest that ERT+MTX treatment may reduce chronic immune activation in IOPD, helping shift the immune landscape toward a more regulated and less inflammatory state.

Age-adjusted multiple regression of the pre-treatment group revealed that higher Glc_4_ was associated with higher naïve cells and lower CM cells (**Supplementary Table 7**). There was no significant association in the whole pre+post cohort, potentially due to the effect of ERT+MTX therapy on proliferating cells in the post-treatment groups, as described above. Together, the maturational and TIGIT profile of CD8^+^ may suggest that untreated IOPD patients may have a tendency toward developing premature T cell immune exhaustion, which is commonly seen in scenarios of chronic inflammation. While further functional studies would be necessary to definitively test this possibility, these findings could suggest that untreated IOPD patients are at risk for dysfunctional T cell responses.

### 4.2 Therapeutic ERT+MTX in LOPD: findings and controversies

The LOPD cohort analyzed in this study represented a significant age span, ranging from 31–77 years of age at the time that they began ERT+MTX. This presents several challenges in analyzing their phenotype, particularly the natural immunological changes that occur during aging (i.e., immunosenescence), and the increasing likelihood that immune phenotype could be influenced by factors other than LOPD, including chronic inflammation or infection. While we made efforts to exclude known confounders, there may be unaccounted factors that influence phenotypes. The LOPD cohort is relatively small (n=6), potentially limiting the ability to generalize to a wider population. An advantage of this group, however, was the ability to analyze phenotypes from the same individuals before and ~6 months after initiating treatment.

Compared to pre-treatment measurements, ERT+MTX treatment in LOPD patients was associated with an decreased frequency of all B cells, as well as a relatively increased percentage of mature B cells and plasmablasts. We also observed an increase in TIGIT^+^ CD4^+^ T cells, which was not accounted for by an increase in the subset of TIGIT^+^ Tregs, Tfh cells, or a specific maturational (naïve/memory) subsets. TIGIT engagement in CD4^+^ cells can mediate inhibitory signals that limit the differentiation and function of subsets such as T helper 1 (Th1) and Th17 cells that are more commonly associated with inflammation and immune activation (53, 54). Thus, the increased presence of TIGIT^+^ CD4^+^ T cells in the post-ERT+MTX LOPD group could signify a therapeutic effect of immunomodulation, and a shift toward a more regulatory (i.e., less activated) T cell population.

The effects of ERT+MTX on LOPD patients were alternately congruent (i.e., increased memory B cells) or different (i.e., increased plasmablasts) compared to observed effects in IOPD. Further, there were no significant correlation between Glc_4_ and immune parameters in LOPD patients that were significant in age-adjusted multiple regression. However, this lack of statistically significant findings does not necessarily imply that ERT+MTX therapy has no impact on patients’ immune phenotypes. As previously outlined, age-related variations in immune responses may have contributed to the observed variability, potentially masking therapy-associated immune changes. The impact of age itself on immune parameters is clear in the multiple significant correlations between age and immune parameters that were revealed upon simple logistic regression (**Supplementary Table 8**). The small number of patients included in this study (n=6) also decreases the likelihood that subtle immunological changes would reach a level of significant detection. Finally, the lack of association between Glc_4_ and immune parameters in LOPD may reflect the much lower levels of glycogen accumulation (and therefore Glc_4_) in this disease compared to IOPD, and therefore effects of ERT on immune parameters may be consequently dampened.

### 4.3 Immune profiling in other ITI regimens suggests a role for regulatory cell types and suppression of type 2 responses in successful immune tolerance.

Although multiple ITI regimens for ERT have been used successfully and clinical and laboratory outcomes have been reported, few studies have performed deep immune phenotyping of treated patients (9, 14, 55–57). We previously reported successful ITI in CRIM-negative IOPD using rituximab plus methotrexate, with or without IVIG, and observed recovery of CD19^+^ B cells after treatment (56). Although detailed immunophenotyping was not performed in that study, prior work in rituximab-treated patients with rheumatoid arthritis and other autoimmune diseases showed reconstitution of the B-cell compartment, with early repopulation of transitional and naïve B cells and delayed recovery of memory subsets (58, 59). In hemophilia, successful ITI for factor inhibitors is associated with increased Bregs and Tregs, as well as increased PD-1 and CTLA-4 expression on T cells (60). IVIG-containing regimens may also contribute to tolerance, potentially through expansion of natural Tregs, although the mechanism remains incompletely defined (61). Recently, we performed in-depth immunophenotyping on IOPD patients who failed immune tolerance after ITI and identified a distinct immune signature associated with developing high sustained anti-rhGAA antibody titers, characterized by a type 2-skewed response with increased Th2 cells and elevated Th2- and proinflammatory cytokines.

### 4.4 Shifting toward type 2 immunity: supporting B cell maturation and production

In two of six LOPD patients (MTX-LOPD3 and MTX-LOPD5) and one of three IOPD patients (MTX-IOPD2), we observed a break in immune tolerance, marked by high antibody titers. CRIM-negative patients are particularly prone to developing anti-rhGAA IgG antibodies because they do not produce GAA naturally, thus limiting their ability to delete autoreactive T and B cells through central tolerance mechanisms. Interestingly, some CRIM-positive patients also develop HSAT despite having endogenous GAA. This finding suggests that additional immunological and physiological factors play a role in shifting the balance from tolerance to an active immune response in HSAT patients. Notably, the MTX-LOPD3 and MTX-LOPD5 patients demonstrated a decrease in Th17 cells and an increase in Th2 cells after ERT+MTX treatment. In addition, we observed a much lower frequency of Th17 in MTX-IOPD2 (post-ERT+MTX) compared to the mean of the unmatched baseline samples. These results are consistent with our prior work on IOPD patients with HSAT, where we identified an increase in circulating Th2 cells and a decrease in Th17 cells as being associated with increased risk for developing HSAT (14). The observed similarities between patients who broke tolerance in the current study and IOPD patients described in our previous study who developed HSAT suggest that these immune characteristics could be useful to predict the development of anti-drug antibodies in both IOPD and LOPD.

In conclusion, this study highlights the complex immunological landscape in patients with Pompe disease receiving ERT and TLD-MTX as an immunomodulatory strategy. Although both LOPD and IOPD are caused by pathogenic variants in *GAA*, they present distinct clinical features, age of onset, and survival rates (1). These differences underscore the need to anticipate distinct immune phenotypes between the two forms. It is essential to note, however, that the small sample size of the LOPD cohort (n=6), and the unmatched samples in the IOPD cohort with a small number in the post-treatment IOPD group (n = 3) limits the statistical power of this analysis and may restrict the generalizability of these findings. Despite these caveats, the significant findings of this study not only suggest that untreated Pompe disease leads to chronic inflammation and that ERT itself may modulate inflammation by reducing glycogen accumulation, but also that the addition of MTX may further modulate chronic inflammation and shift immune cell profiles toward a more regulated state in both IOPD and LOPD patients, potentially improving clinical outcomes.

## Data Availability

The original contributions presented in the study are included in the article/**Supplementary Material**. Further inquiries can be directed to the corresponding authors.
