# Supplementary material for: Enzyme replacement therapy (ERT) combined with transient low-dose methotrexate (TLD-MTX) results in age- and disease-dependent immune profile changes in Infantile- vs. late-onset Pompe disease patients
Source: Front Immunol. 2026 Sep 4;17:1843379. doi: 10.3389/fimmu.2026.1843379 (PMC13585601; doi:10.3389/fimmu.2026.1843379)
Supplement: Supplementary file 1 [file Supplementaryfile1.zip › Supplementary Figure 6.docx]

**Supplementary Figure 6: TIGIT+ cell subset analysis**

**A) IOPD: CD8+ TIGIT+ Cells**

IOPD: Maturation subsets of CD8+ TIGIT+ cells

**B) IOPD: CD4+TIGIT+ cells**

IOPD: CD4+TIGIT+ cells - %Tregs and %TFH

IOPD: Maturation subsets of CD4+TIGIT+ Cells

**C) LOPD: CD8+TIGIT+ cells**

LOPD – Maturation subsets of CD8+TIGIT+ cells

**D) LOPD: CD4+TIGIT+ Cells**

LOPD: CD4+TIGIT+ cells - %Tregs and %TFH

LOPD: Maturation subsets of CD4+TIGIT+ Cells
