# Supplementary material for: Enzyme replacement therapy (ERT) combined with transient low-dose methotrexate (TLD-MTX) results in age- and disease-dependent immune profile changes in Infantile- vs. late-onset Pompe disease patients
Source: Front Immunol. 2026 Sep 4;17:1843379. doi: 10.3389/fimmu.2026.1843379 (PMC13585601; doi:10.3389/fimmu.2026.1843379)
Supplement: Supplementary file 1 [file Supplementaryfile1.zip › Supplementary Figure 8.docx]

**Supplementary Figure 8: Analysis of PD-1 expression on TIGIT+ T cells**

A) **Sample gating of PD-1 gating on TIGIT+ CD4 and CD8 cells from a representative LOPD patient**

B) **IOPD: comparison of TIGIT+PD-1+ cells in IOPD patients untreated and treated with ERT-MTX**

C) **LOPD: comparison of TIGIT+PD-1+ cells in LOPD patients pre- and post-treatment with ERT-MTX**
