# Supplementary material for: Enzyme replacement therapy (ERT) combined with transient low-dose methotrexate (TLD-MTX) results in age- and disease-dependent immune profile changes in Infantile- vs. late-onset Pompe disease patients
Source: Front Immunol. 2026 Sep 4;17:1843379. doi: 10.3389/fimmu.2026.1843379 (PMC13585601; doi:10.3389/fimmu.2026.1843379)
Supplement: Supplementary file 1 [file Supplementaryfile1.zip › Supplementary Materials Guide and Captions.docx]

**Enzyme replacement therapy (ERT) combined with transient low-dose methotrexate (TLD-MTX) results in age- and disease-dependent immune profile changes in Infantile- vs. Late-Onset Pompe Disease patients**

*Parisa Amirifar^1^, PhD, Ankit K. Desai^1^, MBBS, Seung-Hye Jung, PhD^1^, Sarah P. Young, PhD^1,2^, P. Brian Smith. MD^3,4^,* *Cheng Lu^1^, Bijan Abar^1^, PhD, Priya S. Kishnani, MD^1*^, Trevor D. Burt, MD^3,5,6,7*^*

*^1^Division of Medical Genetics, Department of Pediatrics, Duke University Health System, Durham, NC, United States*

*^2^Biochemical Genetics Laboratory, Duke University Health System, Durham, NC, United States*

*^3^Division of Neonatology, Department of Pediatrics, Duke University School of Medicine, Durham, NC, United States*

*^4^Duke Clinical Research Institute, Durham, NC, United States*

*^5^Children’s Health and Discovery Initiative, Duke University School of Medicine, Durham, NC, United States*

*^6^Department of Integrative Immunobiology, Duke University School of. Medicine, Durham, NC, United States*

*^7^Duke Molecular Physiology Institute, Duke University, Durham, NC, United States*

***Corresponding Authors**:

Priya S. Kishnani, MD

*Email: Priya.kishnani@duke.edu*

Trevor D. Burt, MD

*Email:* [*trevor.burt@duke.edu*](mailto:trevor.burt@duke.edu)

**Supplementary Table 1.** Flow Cytometry Antibodies.

**Supplementary Table 2.** Summary of baseline and follow-up laboratory findings in LOPD and IOPD patients.

**Supplementary Table 3.** P values from the Mann-Whitney test comparing immune markers in unmatched ERT+MTX-untreated and -treated samples in IOPD patients.

**Supplementary Table 4.** P values from the Wilcoxon signed-rank test comparing immune markers in pre- and post-ERT+MTX in LOPD patients.

**Supplementary Table 5.** Simple linear regression analysis of age at sample collection (months) versus immune cell subsets in all IOPD patients, ERT+MTX-untreated and -treated groups.

**Supplementary Table 6.** Simple linear regression analysis of Glc_4_ levels versus immune cell subsets in all IOPD patients, ERT+MTX-untreated and -treated groups.

**Supplementary Table 7.** Multiple linear regression analysis of age-adjusted Glc_4_ levels and immune cell subsets in IOPD patients.

**Supplementary Table 8.** Simple linear regression analysis of age and Glc_4_ levels with immune cell subsets in pre- and post-ERT+MTX LOPD patients.

**Supplementary Table 9.** Multiple linear regression analysis of age-adjusted Glc_4_ levels and immune cell subsets in LOPD patients.

**Supplementary Table 10.** Immune parameter values from adult PBMC used as technical biological control. Tables outline the mean values from n=3 replicates of the same healthy adult donor used as a technical control for gating in all flow cytometry runs as a gating standard to minimize batch to batch variability in gating. Values represent the percent of cells within the gate as a percentage of the parent gate. See gating scheme in Supplemental Figures 3 and 4 for reference gating.

**Supplementary Figure 1. Lifetime average enzyme replacement therapy (ERT) dosing.** Timeline of ERT dosing regimens for individual IOPD and LOPD patients, showing the duration (months) at each administered ERT dose and dosing frequency (weekly [W] or every other week [EOW]). Bars represent cumulative time on each ERT dose for each patient.

**Supplementary Figure 2.** **Longitudinal trends in serum biomarkers among LOPD and IOPD patients treated with ERT+MTX.** (A) Serum creatine kinase (CK), (B) aspartate transaminase (AST), and (C) alanine transaminase (ALT) levels were measured at baseline and multiple follow-up time points (up to 36 months) in LOPD and IOPD patients receiving ERT+MTX. Each colored line represents an individual patient over time (LOPD = 6 patients and IOPD = 3 patients).

**Supplementary Figure 3. Representative flow cytometry plots and gating strategies.**

(A) Progressive gating strategy of PBMCs to identify T cell subsets, including: sub-gating of CD4^+^ and CD8^+^ T cells to identify CCR7^+^CD45RA^+^ Naïve, CCR7^+^CD45RA^+^ central memory (CM), CCR7^-^CD45RA^+^ effector memory (EM), CCR7-CD45RA+ terminal effector memory (TEMRA) cells; sub-gating of CD4^+^ cells to identify CD38^+^/hi and activated CD38^+^/hiHLA-DR^+^ cells, Th subsets (Th1 enriched CCR6^-^CXCR3^+^ cells, Th2 enriched CCR6^-^CXCR3^-^ cells, and Th17 enriched CCR6^+^CXCR3^-^ cells), CD45RA^-^CXCR5^+^ T follicular helper (Tfh) cells, CD45RA^+^ CXCR5^+^CD25^+^CD127^-^ T follicular regulatory (Tfr), and CD25^+^CD127^-^ T regulatory (Treg) cells. (B) Progressive gating strategy of PBMCs to identify B cell, NK cell, and myeloid cell subsets, including: subgating of HLA-DR^+^CD14^-^ dendritic cells (DCs) to identify DC cell subsets (CD11c^+^CD123^-^ myeloid dendritic cells (mDCs) and HLA-DR^+^CD14^-^CD11c^-^CD123^+^ plasmacytoid dendritic cells (pDCs)); subgating of HLA-DR^+^CD14^+^ monocytes subsets to identify monocyte subsets (HLA-DR^+^CD14^+^CD14^+^CD16^-^ classical monocytes, and HLA-DR^+^CD14^+^CD14^+^CD16^+^ non-classical monocytes and intermediate monocytes – labelled non-classical on figure for space limitation); subgating of CD56^+^ NK cells to identify NK cell subsets (CD16^+^CD56^+^, CD16^+^CD56^++^, and CD16^-^CD56^++^ NK cells); and sub-gating of CD19^+^ B cells to identify CD19^+^ IgD^+^CD27^-^ naïve, CD19^+^ IgD^+^CD27^+^ unswitched memory, CD19^+^ IgD-CD27^+^ mature memory, and CD19^+^CD20^+^CD38^++^CD24^++^ transitional B cells as well as CD19^+^CD27^+^ IgD^-^CD20^-^CD38^++^ plasmablasts.

**Supplementary Figure 4. Graphical representation of transient low dose methotrexate regimen.**

**Supplementary Figure 5. Maturation subsets of CD4 and CD8 cells from IOPD and LOPD patient before and after treatment with ERT-MTX**

Comparison of T cell subpopulations (as a percent of CD4^+^ or CD8^+^ cells) in (A) IOPD that were untreated or treated with ERT-MTX and (B) LOPD patients pre- and post-treatment with ERT-MTX. Pan CD4^+^ and CD8^+^ cells were gated on CD45RA and CCR7 as outlined in Supplementary Figure 3 and defined as Naïve (CCR7^+^CD45RA^+^), Central Memory (CCR7^+^CD45RA^-^), Effector Memory (CCR7^-^CD45RA^-^), or TEMRA (CCR7^-^CD45RA^+^). Statistical comparisons were performed. Using the Mann-Whitney test for IOPD samples and Wilcoxon signed-rank test for LOPD. *P<0.05, “ns” indicates non-significant difference.

**Supplementary Figure 6. Cell subsets of TIGIT^+^ CD4 and CD8 cells from IOPD and LOPD patient before and after treatment with ERT-MTX**

Comparison of TIGIT^+^ T cell subpopulations (as a percent of CD4^+^TIGIT^+^ or CD8^+^ TIGIT^+^ cells) in (A-B) IOPD that were untreated or treated with ERT-MTX and (B-C) LOPD patients pre- and post-treatment with ERT-MTX. CD4^+^TIGIT^+^ and CD8^+^TIGIT^+^ cells were gated on CD45RA and CCR7 as outlined in Supplementary Figure 3 and defined as Naïve (CCR7^+^CD45RA^+^), Central Memory (CCR7^+^CD45RA^-^), Effector Memory (CCR7^-^CD45RA^-^), or TEMRA (CCR7^-^CD45RA^+^). Tregs were defined as CD4^+^CD127^low^CD25^hi^ and TFH were defines as CD4^+^CD45RA^-^CCR5^+^. Statistical comparisons were performed. Using the Mann-Whitney test for IOPD samples and Wilcoxon signed-rank test for LOPD. *P<0.05 was considered statistically significant.

**Supplementary Figure 7. Cell subsets of regulatory T cells (Tregs) IOPD and LOPD patients before and after treatment with ERT-MTX**

Comparison of CD4^+^CD25^hi^CD127^low^ Tregs (as a percent of CD4^+^ cells) in (A) IOPD that were untreated or treated with ERT-MTX and (B) LOPD patients pre- and post-treatment with ERT-MTX. CD4 cells were gated on CD25 and CD127 as outlined in Supplementary Figure 3 and Tregs were defined as CD4^+^CD127^low^CD25^hi^. Statistical comparisons were performed. Using the Mann-Whitney test for IOPD samples and Wilcoxon signed-rank test for LOPD. *P<0.05 was considered statistically significant.

**Supplementary Figure 8. Analysis of PD-1 expression on TIGIT+ T cells in IOPD and LOPD patient before and after treatment with ERT-MTX**

(A) Example of flow cytometry gating of TIGIT^+^ population from CD4^+^ and CD8^+^ cells from a representative LOPD patient. (B-C) Comparison of %TIGIT^+^PD-1^+^ (as a percent of TIGIT^+^CD4^+^ and TIGIT^+^CD8^+^ cells) in (B) IOPD that were untreated or treated with ERT-MTX and (C) LOPD patients pre- and post-treatment with ERT-MTX. Statistical comparisons were performed. Using the Mann-Whitney test for IOPD samples and Wilcoxon signed-rank test for LOPD. *P<0.05 was considered statistically significant.
