# Supplementary material for: Enzyme replacement therapy (ERT) combined with transient low-dose methotrexate (TLD-MTX) results in age- and disease-dependent immune profile changes in Infantile- vs. late-onset Pompe disease patients
Source: Front Immunol. 2026 Sep 4;17:1843379. doi: 10.3389/fimmu.2026.1843379 (PMC13585601; doi:10.3389/fimmu.2026.1843379)
Supplement: Supplementary file 1 [file Supplementaryfile1.zip › Supplementary Table 1.docx]

| **Antibody** | **Fluor** | **Supplier** | **Cat#** | **Clone** |
| --- | --- | --- | --- | --- |
| TIGIT | APC | Biolegend | 372706 | A15153G |
| CD3 | AlexaFluor 700 | Biolegend | 344822 | SK7 |
| CD8 | APC-Cy7 | Biolegend | 344714 | SK1 |
| CD25 | BB515 | BD | 564467 | 2A3 |
| CD45RA | PerCP-Cy5.5 | Biolegend | 304122 | HI100 |
| CD38 | BV421 | Biolegend | 303526 | HIT2 |
| Zombie | Aqua Live/Dead | Biolegend | N/A | N/A |
| CCR4 | BV605 | Biolegend | 359418 | L291H4 |
| CD127 | BV605 | Biolegend | 351325 | A019D5 |
| PD-1 | BV750 | Biolegend | 329966 | EH12.2H7 |
| CCR6 | BV785 | Biolegend | 353422 | G034E3 |
| CXCR3 | PE | BD | 557815 | 1C6/CXCR3 |
| CCR7 | PE-CF594 | BD | 562381 | 150503 |
| CD19 | PE-Cy5 | Biolegend | 302210 | HIB19 |
| CXCR5 | PE-Cy7 | Biolegend | 356924 | J252D4 |
| CD14 | BUV395 | BD | 563561 | MOP9 |
| CD56 | BUV563 | BD | 612298 | NCAM16.2 |
| HLA-DR | BUV661 | BD | 612891 | G46-6 |
| CD39 | BUV737 | BD | 612852 | TU66 |
| CD4 | BUV805 | BD | 612887 | SK3 |
| CD20 | FITC | Biolegend | 302230 | 2H7 |
| CD19 | PerCP-Cy5.5 | Biolegend | 302230 | HIB19 |
| CD38 | BV421 | Biolegend | 356618 | HB-7 |
| Zombie | Aqua | Biolegend | N/A | N/A |
| CD24 | BV650 | BD | 563720 | ML5 |
| CD27 | BV750 | BD | 302856 | O323 |
| CD16 | BV785 | Biolegend | 302046 | 3G8 |
| CD14 | BUV395 | BD | 563561 | MOP9 |
| CD56 | BUV737 | BD | 612787 | NCAM16.2 |
| HLA-DR | APC | Biolegend | 307610 | L243 |
| CD123 | AlexaFluor 700 | BD | 306040 | 6H6 |
| CD3 | APC-Cy7 | Biolegend | 345818 | SK7 |
| CD138 | PE | Biolegend | 356504 | MI15 |
| CD11c | PE-Cy5 | BD | 551077 | B-ly6 |
| IgD | PE-Cy7 | Biolegend | 348210 | IA6-2 |
