# Supplementary material for: Enzyme replacement therapy (ERT) combined with transient low-dose methotrexate (TLD-MTX) results in age- and disease-dependent immune profile changes in Infantile- vs. late-onset Pompe disease patients
Source: Front Immunol. 2026 Sep 4;17:1843379. doi: 10.3389/fimmu.2026.1843379 (PMC13585601; doi:10.3389/fimmu.2026.1843379)
Supplement: Supplementary file 1 [file Supplementaryfile1.zip › Supplementary Table 10.docx]

**Supplementary Table 10:** **Batch control (healthy adult) values from single healthy donor.** Based on the average of a minimum of 3 single sample runs from the same individual donor during each flow cytometry run for the current project. These samples were used to set gates and to ensure consistency between experiments. Values reported are the mean of the parent gate (e.g., All CD3+ T cells are reported as a % of the parent “Lymphocytes” gate – see gating scheme in Supplemental Figure 3 and 4 for reference). Gates reflect the same parameter gating reported in Table 2.

| **T cells** |  |
| --- | --- |
| **Parameter** | **Mean %** |
| All CD3+ T cells | 78.88 |
| All CD4+ T cells | 64.63 |
| All CD8 T cells | 31.85 |
| CD4+ Central Memory | 44.08 |
| CD4+ Naïve | 33.00 |
| CD4+ TEMRA | 4.51 |
| CD4+ Effector Memory | 18.41 |
| CD4+ {PD-1+ | 0.91 |
| CD4+ TIGIT+ | 2.07 |
| CD3+ CD38hi | 42.75 |
| CD4+ Activated | 0.73 |
| CD4+ TFH | 7.28 |
| CD4+ TFr | 2.69 |
| CD4+ Tregs | 5.27 |
| CD4+ Th17 | 15.30 |
| CD4+ Th1 | 29.66 |
| CD4+ Th2 | 48.59 |
| CD8+ Central Memroy | 4.61 |
| CD9+ Naïve | 29.88 |
| CD9+ TEMRA | 26.05 |
| CD8+ Effector Memroy | 39.46 |
| CD8+ PD1+ | 1.27 |
| CD8+ TIGIT+ | 12.61 |

| **B cells** |  |
| --- | --- |
| **Parameter** | **Mean %** |
| All CD19+ B cells | 4.34 |
| Switched Memory B cells | 19.63 |
| Plamsablasts | 1.64 |
| Naïve B cells | 65.69 |
| Transitional B cells | 2.75 |
| Unswitched Memory B cells | 7.60 |

| **Innate Cells** |  |
| --- | --- |
| **Parameter** | **Mean %** |
| All CD14+ Monocytes | 84.00 |
| CD14+CD16- Classical Monocytes | 96.40 |
| CD16+ Monocytes | 2.96 |
| All Dendritic Cells | 5.36 |
| Myeloid Dendritic Cells | 69.91 |
| Plasmacytoid Dendritic Cells | 3.66 |
| All NK cells | 7.58 |
| CD16+CD56+ NK Cells | 80.05 |
| CD16+CD56++ NK Cells | 6.59 |
| CD16-CD56++ NK Cells | 8.26 |
