# Supplementary material for: Enzyme replacement therapy (ERT) combined with transient low-dose methotrexate (TLD-MTX) results in age- and disease-dependent immune profile changes in Infantile- vs. late-onset Pompe disease patients
Source: Front Immunol. 2026 Sep 4;17:1843379. doi: 10.3389/fimmu.2026.1843379 (PMC13585601; doi:10.3389/fimmu.2026.1843379)
Supplement: Supplementary file 1 [file Supplementaryfile1.zip › Supplementary Table 2.docx]

| Age and Biomarkers * | Time points | MTX-LOPD1 | MTX-LOPD2 | MTX-LOPD3 | MTX-LOPD4 | MTX-LOPD5 | MTX-LOPD6 | MTX-IOPD1 | MTX-IOPD2 | MTX-IOPD3 |
| --- | --- | --- | --- | --- | --- | --- | --- | --- | --- | --- |
| Age  (Year for LOPD and Month for IOPD pts) | Baseline | 62.82 | 38.21 | 63.25 | 77.65 | 31.33 | 31.98 | - | - |  |
|  | 0-month | 62.8 | 38.2 | 63.3 | 77.7 | 31.3 | 32.0 | 3.40 | 3.90 | 6.67 |
|  | 1-month | 62.9 | 38.3 | 63.4 | 77.8 | 31.4 | 32.1 | 4.40 | 4.90 | 7.67 |
|  | 2-month | 63.0 | 38.4 | 63.5 | 77.9 | 31.5 | 32.2 | 5.40 | 5.90 | 8.67 |
|  | 3-month | 63.0 | 38.5 | 63.5 | 78.0 | 31.6 | 32.2 | 6.40 | 6.90 | 9.67 |
|  | 4-month | 63.1 | 38.5 | 63.6 | 78.0 | 31.6 | 32.3 | 7.40 | 7.90 | 10.67 |
|  | 5-month | 63.2 | 38.6 | 63.7 | 78.1 | 31.7 | 32.4 | 8.40 | 8.90 | 11.67 |
|  | 6-month | 63.3 | 38.7 | 63.8 | 78.2 | 31.8 | 32.5 | 9.40 | 9.90 | 12.67 |
|  | 7-month | 63.4 | 38.8 | 63.9 | 78.3 | 31.9 | 32.6 | 10.40 | 10.90 | 13.67 |
|  | 8-month | 63.5 | 38.9 | 64.0 | 78.4 | 32.0 | 32.7 | 11.40 | 11.90 | 14.67 |
|  | 9-month | 63.5 | 39.0 | 64.0 | 78.5 | 32.0 | 32.8 | 12.40 | 12.90 | 15.67 |
|  | 10-month | 63.6 | 39.0 | 64.1 | 78.5 | 32.1 | 32.8 | 13.40 | 13.90 | 16.67 |
|  | 11-month | 63.7 | 39.1 | 64.2 | 78.6 | 32.2 | 32.9 | 14.40 | 14.90 | 17.67 |
|  | 12-month | 63.8 | 39.2 | 64.3 | 78.7 | 32.3 | 33.0 | 15.40 | 15.90 | 18.67 |
|  | 18-month | 64.3 | 39.7 | 64.8 | 79.2 | 32.8 | 33.5 | 21.40 | 21.90 | 24.67 |
|  | 24-month | 64.8 | 40.2 | 65.3 | 79.7 | 33.3 | 34.0 | 27.40 | 27.90 | 30.67 |
|  | 30-month | 65.3 | 40.7 | 65.8 | 80.2 | 33.8 | 34.5 | 33.40 | 33.90 | 36.67 |
|  | 36-month | 65.8 | 41.2 | 66.3 | 80.7 | 34.3 | 35.0 | 39.40 | 39.90 | 42.67 |
| anti-GAA Ab  Normal range <12800 | Baseline | - | - | - | - | - | - | 0 | 0 | 0 |
|  | 0-month | - | 0 | - | - | - | - | - | - | - |
|  | 1-month | 0 | 100 | 0 | - | - | - | - | - | 0 |
|  | 2-month | - | 200 | 400 | - | - | - | - | 400 | 800 |
|  | 3-month | 800 | 200 | 3200 | - | - | - | - | 12800 | 6400 |
|  | 4-month | 800 | - | - | - | - | 0 | - | 12800 | 12800 |
|  | 5-month | - | - | 12800 |  | - | - | 200 | 6400 | 3200 |
|  | 6-month | 400 | 200 | - | 6400 | 12800 | - | 200 | 12800 | - |
|  | 7-month | 400 | - | - | - | 25600 | - | 100 | 25600 | - |
|  | 8-month | - | - | - | - | 25600 | - | - | 12800 | - |
|  | 9-month | - | 800 | - | - | - | - | - | 6400 | - |
|  | 10-month | - | - | 12800 | - | 51200 | - | - | - | - |
|  | 11-month | - | - | 12800 | - | 51200 | - | - | - | - |
|  | 12-month | - | 1600 | 12800 | 1600 | 51200 | - | 0 | - | - |
|  | 18-month | 400 | 400 | 3200 | 1600 | 12800 | - | - | - | - |
|  | 24-month | 800 | 800 | 6400 | 1600 | 6400 | - | - | - | - |
|  | 30-month | 800 | - | 1600 | 6400 | 6400 | 400 | - | - | - |
|  | 36-month | 400 | - | 3200 | 3200 | - | - | - | - | - |
| CK (U/L) | Baseline | 110 | 928 | 324 | 301 | 837 | 639 | 461 | 649 | 665 |
|  | 0-month | 85 | - | 339 | - | 559 | - | 366 | 730 | - |
|  | 1-month | 113 | - | 354 | - | 879 | - | 409 | - | - |
|  | 2-month | 73 | - | 420 | - | 480 | - | 411 | 991 | - |
|  | 3-month | 101 | 1202 | 340 | - | - | - | 439 | 1187 | - |
|  | 4-month | - | - | - | - | 694 | 459 | 364 | - | - |
|  | 5-month | - | - | 311 | - | 785 | - | - | - | - |
|  | 6-month | - | - | - | 211 | 844 | - | 818 | 1169 | - |
|  | 7-month | - | - | - | - |  | - | 889 | - |  |
|  | 8-month | - | - | - | - | - | - | 505 | - | - |
|  | 9-month | - | 645 | - | - | - | - | 418 | 1454 | - |
|  | 10-month | 85 | - | - | - | - | - | 745 | - | - |
|  | 11-month | - | - | - | - | 658 | - | 728 | - | - |
|  | 12-month | - | 545 | - | - | 1219 | 562 | 669 | 981 | - |
|  | 18-month | 115 | - | 294 | 282 | 831 | 589 | 908 | - | - |
|  | 24-month | 105 | 483 | 357 | 248 | 831 | 381 | 527 | - | - |
|  | 30-month | 90 | - | 234 | 171 | 1091 | 434 | 906 | - | - |
|  | 36-month | 102 | 544 | 230 | 150 | - | - | - | - | - |
| Glc4 (mmol/mol Cr) | Baseline | 4 | 12.2 | 5.8 | 2.9 | 7.3 | 6.8 | 26.5 | 35.8 | 42.7 |
|  | 0-month | - | - | - | - | 4 | - | - | - | - |
|  | 1-month | - | 11.7 | 5.3 | - | 4.9 | 3.9 | 14.4 | 21.6 | - |
|  | 2-month | 3.4 | 9.6 | 5 | - | 5.1 | - | 17 | 20.7 | 25.2 |
|  | 3-month | - | - | 3.8 | - | 4.6 | 6 | 11.2 | 13.2 | 20 |
|  | 4-month | 4 | - | - | - | 6.3 | - | 22 | 20 | 21.3 |
|  | 5-month | - | - | - | - | 4.7 | - | - | 25.1 | 20.7 |
|  | 6-month | - | - | 4.9 | 2 | 5.8 | - | - | 14.6 | 22.3 |
|  | 7-month | - | - | - | - | 4.8 | - | - | 17.3 | 22 |
|  | 8-month | - | - | - | - | - | - | - | 22.6 | 18.7 |
|  | 9-month |  | 7.9 | - | - | - | - | 10.7 | 20.1 | 17.5 |
|  | 10-month | 2.4 | - | - | - | - | - | - | 23.1 | 17.1 |
|  | 11-month | - | - | - | - | 4.6 | - | - | - | - |
|  | 12-month | - | 5.3 | 4.3 | 3 | 11.2 | 5.1 | 14.8 | - | 20.7 |
|  | 18-month | 2.9 | - | 3.6 | 4.2 | 10.3 | - | 17.4 | - | 18.7 |
|  | 24-month | 2.7 | 7.4 | 5.3 | 3.8 | - | 5 | 32.9 | - | - |
|  | 30-month | 3 | - | 3.2 | 3.6 | 9.7 | - | 21.9 | - | - |
|  | 36-month | 2.5 | - | 4.6 | 2.6 | - | - | - | - | - |
| AST (U/L) | Baseline | 43 | 93 | 46 | 38 | 97 | 77 | 102 | 268 | 598 |
|  | 1-month | 36 | - | 43 | - | 87 | - | 115 | - | - |
|  | 3-month | 32 | 97 | 42 | - | - | - | 81 | 329 | - |
|  | 6-month | - | - | 41 | 27 | 76 | - | 157 | 274 | - |
|  | 9-month | 25 | 59 | - | - | - |  | 150 | 368 | - |
|  | 12-month | - | 65 | 39 | - | 79 | - | 164 | 446 | 422 |
|  | 18-month | 30 | 52 | 40 | 40 | 116 | 49 | 162 | - | - |
|  | 24-month | 23 | - | 40 | 33 | 87 | 43 | 281 | - | - |
|  | 30-month | 27 | - | 37 | 23 | - | 50 | 99 | - | - |
|  | 36-month | 27 | 63 | 35 | 23 | - | - | 145 | - | - |
| ALT (U/L) | Baseline | 39 | 86 | 54 | 32 | 111 | 84 | 48 | 111 | 205 |
|  | 1-month | 31 | - | 50 | - | 101 | - | 88 | - | - |
|  | 3-month | 28 | 70 | 42 | - | - | - | 66 | 111 | - |
|  | 6-month | - | - | 47 | 25 | 101 | - | 73 | 124 | - |
|  | 9-month | 26 | 54 | - | - | - | - | 79 | 151 | - |
|  | 12-month | - | 57 | 45 | - | 102 | - | 85 | 136 | 164 |
|  | 18-month | 35 | 70 | 46 | - | 96 | 55 | - | - | - |
|  | 24-month | 28 | - | 47 | 30 | 129 | 55 | - | - | - |
|  | 30-month | 27 |  | 42 | 22 | 111 | 53 | - | - | - |
|  | 36-month | 30 | 93 | 37 | 17 | - | - | - | - | - |

CK: Creatine Kinase, AST: Aspartate Aminotransferase, ALT: Alanine Aminotransferase

*Reference ranges:

**CK**

< 10 Years:	70 - 320 U/L; 10 - 19 Years: 70 - 250 U/L; > 19 Years 30 - 220 U/L (females), 60 - 270 U/L (males)

**Glc_4_**

0 to 6 months: <20 mmol/mol creatinine, 6 to 12 months: <14 mmol/mol creatinine, 1 to 3 years: <8.3 mmol/mol creatinine, > 3 years of age: 3.0 mmol/mol creatinine.

**AST**

< 1 Year: 30 - 120 U/L; > 1 Year 15 - 41 U/L

**ALT**

< 1 Year: 5 - 45 U/L; 1 - 3 Years: 5 - 40 U/L; 3 - 11 Years: 8 - 40 U/L; > 11 Years: 10 - 39 U/L (females), 15 - 50 U/L (males)
