# Supplementary material for: Enzyme replacement therapy (ERT) combined with transient low-dose methotrexate (TLD-MTX) results in age- and disease-dependent immune profile changes in Infantile- vs. late-onset Pompe disease patients
Source: Front Immunol. 2026 Sep 4;17:1843379. doi: 10.3389/fimmu.2026.1843379 (PMC13585601; doi:10.3389/fimmu.2026.1843379)
Supplement: Supplementary file 1 [file Supplementaryfile1.zip › Supplementary Table 3.docx]

| **Immune markers** | **Two-sample Wilcoxon rank-sum (Mann–Whitney) test** |
| --- | --- |
|  | **ERT+MTX-untreated vs -treated (P value)** |
| **All CD14+ monocytes** | 0.0180 |
| **Classical monocytes** | 0.0178 |
| **CD16+ monocytes (non-classical + intermediate)** | 0.0280 |
| **All Dendritic cells (DCs)** | 0.0278 |
| **Myeloid Dendritic Cells (mDC; % DCs)** | 0.8658 |
| **Plasmacytoid Dendritic Cells (pDC; % DCs)** | 0.1763 |
| **All B cells** | 0.3098 |
| **Memory B cells** | 0.0422 |
| **Plasmablasts** | 0.0425 |
| **Naïve B cells** | 0.0901 |
| **Transitional B cells** | 0.1282 |
| **Unswitched Memory B cells** | 0.0516 |
| **NK Cells** | 0.3105 |
| **CD16+CD56+ NK Cells** | 0.6121 |
| **CD16+CD56++ NK Cells** | 0.4462 |
| **CD16-CD56++ NK Cells** | 0.4990 |
| **All CD3+ T cells** | 0.3105 |
| **CD4+ T cells** | 0.7353 |
| **CD8+ T cells** | 0.4353 |
| **CD4+ Central Memory** | 0.0422 |
| **CD4+ Naive** | 0.3105 |
| **CD4+ Terminal Effector (TEMRA)** | 0.1277 |
| **CD4+ Effector Memory** | 0.3105 |
| **CD4+ PD-1+** | 0.9326 |
| **CD4+ TIGIT +** | 0.3980 |
| **CD4+ CD38hi** | 0.1757 |
| **CD4+ Activated (HLADR+CD38hi)** | 0.6722 |
| **CD4+ Tfh Cells** | 0.7353 |
| **CD4+ Tfr Cells** | 0.0905 |
| **Treg Cells (%CD25+CD127low)** | 0.8658 |
| **Th17** | 0.3980 |
| **Th1** | 0.3980 |
| **Th2** | 0.3980 |
| **CD8+ Central Memory** | 0.0280 |
| **CD8+ Naive** | 0.0630 |
| **CD8+ Terminal Effector (TEMRA)** | 0.2367 |
| **CD8+ Effector Memory** | 1.000 |
| **CD8+ PD-1 +** | 0.7353 |
| **CD8+ TIGIT+** | 0.0112 |
