# Supplementary material for: Enzyme replacement therapy (ERT) combined with transient low-dose methotrexate (TLD-MTX) results in age- and disease-dependent immune profile changes in Infantile- vs. late-onset Pompe disease patients
Source: Front Immunol. 2026 Sep 4;17:1843379. doi: 10.3389/fimmu.2026.1843379 (PMC13585601; doi:10.3389/fimmu.2026.1843379)
Supplement: Supplementary file 1 [file Supplementaryfile1.zip › Supplementary Table 5.docx]

| **Immune markers** | **Simple Linear Regression (age vs immune markers)** | | | | | | | | | | | | |
| --- | --- | --- | --- | --- | --- | --- | --- | --- | --- | --- | --- | --- | --- |
|  | **All IOPD patients** | | | | **ERT+MTX-untreated IOPD patients** | | | | **ERT+MTX-treated IOPD patients** | | | | |
|  | **Coefficient** | **P value** | **95% conf. low** | **95% conf. high** | **Coefficient** | **P value** | **95% conf. low** | **95% conf. high** | **Coefficient** | **P value** | **95% conf. low** | **95% conf. high** |  |
| **All CD14+ monocytes** | 15.371 | 0.320 | -17.112 | 47.855 | 3.002 | 0.920 | -64.091 | 70.095 | 7.967 | 0.416 | -69.457 | 85.390 |  |
| **Classical monocytes** | 0.729 | 0.896 | -11.299 | 12.757 | -15.430 | 0.063 | -31.938 | 1.078 | -1.823 | 0.596 | -33.280 | 29.634 |  |
| **CD16+ monocytes (non-classical + intermediate)** | 1.468 | 0.764 | -9.022 | 11.958 | 16.444 | 0.030 | 2.013 | 30.874 | 2.053 | 0.546 | -28.124 | 32.230 |  |
| **All Dendritic cells (DCs)** | -6.737 | 0.463 | -26.242 | 12.767 | -4.245 | 0.815 | -44.656 | 36.165 | 1.980 | 0.468 | -20.802 | 24.762 |  |
| **Myeloid Dendritic Cells (mDC; % DCs)** | 12.691 | 0.361 | -16.589 | 41.970 | 47.043 | 0.072 | -5.220 | 99.307 | -8.709 | 0.541 | -134.538 | 117.119 |  |
| **Plasmacytoid Dendritic Cells (pDC; % DCs)** | -9.447 | 0.119 | -21.730 | 2.837 | -13.812 | 0.246 | -39.246 | 11.622 | -2.740 | 0.584 | -48.243 | 42.763 |  |
| **All B cells** | 11.473 | 0.020 | 2.188 | 20.759 | 9.195 | 0.251 | -7.949 | 26.339 | 14.672 | 0.435 | -137.218 | 166.563 |  |
| **Memory B cells** | 1.924 | 0.108 | -0.492 | 4.340 | 3.437 | 0.024 | 0.572 | 6.303 | -2.237 | 0.339 | -19.002 | 14.528 |  |
| **Plasmablasts** | -12.914 | 0.028 | -24.116 | -1.712 | -22.266 | 0.028 | -41.446 | -3.086 | 1.189 | 0.793 | -43.692 | 46.070 |  |
| **Naïve B cells** | -2.499 | 0.431 | -9.233 | 4.236 | -7.916 | 0.150 | -19.370 | 3.539 | 5.992 | 0.279 | -29.672 | 41.655 |  |
| **Transitional B cells** | -12.063 | 0.252 | -34.028 | 9.903 | -26.380 | 0.178 | -67.621 | 14.861 | 10.470 | 0.307 | -59.035 | 79.974 |  |
| **Unswitched Memory B cells** | 0.625 | 0.194 | -0.370 | 1.621 | 0.831 | 0.178 | -0.466 | 2.127 | -0.654 | 0.693 | -16.516 | 15.209 |  |
| **NK Cells** | -0.259 | 0.914 | -5.397 | 4.879 | 3.768 | 0.402 | -6.059 | 13.596 | 0.240 | 0.922 | -24.505 | 24.985 |  |
| **CD16+CD56+ NK Cells** | 13.731 | 0.314 | -14.888 | 42.350 | 13.776 | 0.550 | -37.087 | 64.639 | 44.064 | 0.060 | -9.279 | 97.407 |  |
| **CD16+CD56++ NK Cells** | 0.204 | 0.818 | -1.707 | 2.116 | 0.151 | 0.910 | -2.831 | 3.132 | 1.948 | 0.579 | -29.851 | 33.746 |  |
| **CD16-CD56++ NK Cells** | -6.744 | 0.393 | -23.436 | 9.949 | -9.460 | 0.540 | -43.504 | 24.583 | -14.144 | 0.284 | -100.224 | 71.936 |  |
| **All CD3+ T cells** | -16.105 | 0.318 | -49.975 | 17.764 | -69.119 | 0.006 | -112.415 | -25.823 | -11.249 | 0.580 | -195.786 | 173.287 |  |
| **CD4+ T cells** | -5.578 | 0.327 | -17.538 | 6.382 | -6.803 | 0.455 | -26.791 | 13.185 | -11.116 | 0.646 | -238.083 | 215.850 |  |
| **CD8+ T cells** | 2.538 | 0.644 | -9.213 | 14.288 | -5.946 | 0.548 | -27.800 | 15.908 | 7.186 | 0.652 | -142.663 | 157.034 |  |
| **CD4+ Central Memory** | -2.756 | 0.520 | -11.894 | 6.381 | 2.874 | 0.710 | -14.301 | 20.049 | 1.392 | 0.391 | -11.075 | 13.859 |  |
| **CD4+ Naive** | -0.154 | 0.971 | -9.447 | 9.138 | -4.267 | 0.588 | -21.718 | 13.184 | -4.576 | 0.601 | -84.804 | 75.652 |  |
| **CD4+ Terminal Effector (TEMRA)** | 1.170 | 0.094 | -0.234 | 2.573 | -0.112 | 0.740 | -0.865 | 0.641 | 1.261 | 0.756 | -38.535 | 41.058 |  |
| **CD4+ Effector Memory** | 1.784 | 0.005 | 0.668 | 2.900 | 1.526 | 0.052 | -0.014 | 3.065 | 1.932 | 0.546 | -26.416 | 30.280 |  |
| **CD4+ PD-1+** | 0.063 | 0.801 | -0.470 | 0.595 | 0.119 | 0.809 | -0.985 | 1.224 | 0.297 | 0.187 | -0.843 | 1.437 |  |
| **CD4+ TIGIT +** | 0.048 | 0.893 | -0.714 | 0.809 | 0.233 | 0.722 | -1.227 | 1.693 | 0.572 | 0.253 | -2.485 | 3.629 |  |
| **CD4+ CD38hi** | -5.676 | 0.001 | -8.320 | -3.031 | -8.388 | 0.001 | -12.346 | -4.430 | -3.886 | 0.486 | -51.133 | 43.360 |  |
| **CD4+ Activated (HLADR+CD38hi)** | 0.256 | 0.341 | -0.310 | 0.822 | 0.049 | 0.922 | -1.081 | 1.179 | 0.509 | 0.465 | -5.288 | 6.307 |  |
| **CD4+ Tfh Cells** | 0.434 | 0.111 | -0.117 | 0.986 | 0.311 | 0.467 | -0.628 | 1.249 | 0.854 | 0.445 | -8.263 | 9.972 |  |
| **CD4+ Tfr Cells** | -1.516 | 0.614 | -7.955 | 4.922 | 5.154 | 0.324 | -6.153 | 16.461 | -0.988 | 0.722 | -27.909 | 25.934 |  |
| **Treg Cells (%CD25+CD127low)** | 1.018 | 0.459 | -1.905 | 3.940 | 2.626 | 0.299 | -2.822 | 8.074 | 0.784 | 0.849 | -40.567 | 42.136 |  |
| **Th17** | 4.078 | 0.001 | 1.986 | 6.169 | 1.649 | 0.146 | -0.710 | 4.007 | 6.010 | 0.335 | -38.331 | 50.352 |  |
| **Th1** | -1.444 | 0.842 | -17.068 | 14.179 | 8.945 | 0.527 | -22.229 | 40.119 | -0.867 | 0.892 | -65.305 | 63.571 |  |
| **Th2** | -3.398 | 0.645 | -19.191 | 12.395 | -10.964 | 0.442 | -42.242 | 20.314 | -6.532 | 0.629 | -132.238 | 119.175 |  |
| **CD8+ Central Memory** | -10.149 | 0.056 | -20.590 | 0.293 | -11.763 | 0.220 | -32.154 | 8.627 | -1.168 | 0.385 | -11.422 | 9.086 |  |
| **CD8+ Naive** | 2.705 | 0.670 | -10.906 | 16.316 | -8.598 | 0.439 | -32.941 | 15.746 | -2.316 | 0.194 | -11.582 | 6.951 |  |
| **CD8+ Terminal Effector (TEMRA)** | 2.915 | 0.002 | 1.277 | 4.553 | 3.587 | 0.023 | 0.642 | 6.533 | 2.384 | 0.477 | -25.763 | 30.531 |  |
| **CD8+ Effector Memory** | 4.536 | 0.179 | -2.420 | 11.492 | 16.747 | 0.002 | 8.365 | 25.129 | 1.137 | 0.343 | -7.495 | 9.770 |  |
| **CD8+ PD-1 +** | 0.368 | 0.184 | -0.203 | 0.938 | -0.106 | 0.823 | -1.161 | 0.950 | 0.964 | 0.006 | 0.857 | 1.071 |  |
| **CD8+TIGIT+** | -0.666 | 0.551 | -3.052 | 1.719 | 1.169 | 0.546 | -3.112 | 5.450 | 0.370 | 0.169 | -0.911 | 1.650 |  |
