# Supplementary material for: Enzyme replacement therapy (ERT) combined with transient low-dose methotrexate (TLD-MTX) results in age- and disease-dependent immune profile changes in Infantile- vs. late-onset Pompe disease patients
Source: Front Immunol. 2026 Sep 4;17:1843379. doi: 10.3389/fimmu.2026.1843379 (PMC13585601; doi:10.3389/fimmu.2026.1843379)
Supplement: Supplementary file 1 [file Supplementaryfile1.zip › Supplementary Table 6.docx]

| **Immune markers** | **Simple Linear Regression (Glc_4_ vs immune markers)** | | | | | | | | | | | |
| --- | --- | --- | --- | --- | --- | --- | --- | --- | --- | --- | --- | --- |
|  | **All IOPD patients** | | | | **ERT+MTX-untreated IOPD patients** | | | | **ERT+MTX-treated IOPD patients** | | | |
|  | **Coefficient** | **P value** | **95% conf. low** | **95% conf. high** | **Coefficient** | **P value** | **95% conf. low** | **95% conf. high** | **Coefficient** | **P value** | **95% conf. low** | **95% conf. high** |
| **All CD14+ monocytes** | -0.338 | 0.295 | -1.014 | 0.339 | -0.175 | 0.669 | -1.084 | 0.734 | 1.675 | 0.771 | -54.995 | 58.345 |
| **Classical monocytes** | -0.281 | 0.004* | -0.451 | -0.112 | -0.225 | 0.045 | -0.442 | -0.007 | -0.875 | 0.591 | -15.730 | 13.980 |
| **CD16+ monocytes (non-classical + intermediate)** | 0.242 | 0.005* | 0.091 | 0.393 | 0.214 | 0.044 | 0.008 | 0.420 | 0.798 | 0.641 | -15.213 | 16.808 |
| **All Dendritic cells (DCs)** | 0.102 | 0.597 | -0.311 | 0.516 | -0.003 | 0.989 | -0.559 | 0.553 | -1.088 | 0.345 | -9.395 | 7.220 |
| **Myeloid Dendritic Cells (mDC; % DCs)** | 0.277 | 0.339 | -0.334 | 0.888 | 0.369 | 0.339 | -0.468 | 1.205 | 5.700 | 0.272 | -27.312 | 38.712 |
| **Plasmacytoid Dendritic Cells (pDC; % DCs)** | -0.074 | 0.578 | -0.359 | 0.210 | -0.194 | 0.233 | -0.541 | 0.153 | 2.005 | 0.229 | -7.568 | 11.578 |
| **All B cells** | 0.035 | 0.765 | -0.215 | 0.285 | 0.137 | 0.207 | -0.093 | 0.368 | -7.453 | 0.378 | -71.330 | 56.425 |
| **Memory B cells** | 0.020 | 0.444 | -0.036 | 0.076 | 0.062 | 0.000* | 0.039 | 0.084 | 0.908 | 0.474 | -9.711 | 11.526 |
| **Plasmablasts** | -0.123 | 0.361 | -0.407 | 0.161 | -0.305 | 0.028 | -0.568 | -0.042 | 1.443 | 0.394 | -11.597 | 14.482 |
| **Naïve B cells** | -0.085 | 0.191 | -0.219 | 0.049 | -0.155 | 0.024 | -0.283 | -0.026 | -2.100 | 0.534 | -31.811 | 27.611 |
| **Transitional B cells** | -0.247 | 0.263 | -0.708 | 0.214 | -0.489 | 0.053 | -0.987 | 0.008 | 1.048 | 0.880 | -68.992 | 71.087 |
| **Unswitched Memory B cells** | -0.007 | 0.500 | -0.029 | 0.015 | 0.003 | 0.700 | -0.016 | 0.023 | 0.658 | 0.120 | -0.938 | 2.253 |
| **NK Cells** | 0.041 | 0.406 | -0.063 | 0.145 | 0.017 | 0.785 | -0.123 | 0.158 | 0.853 | 0.265 | -3.934 | 5.639 |
| **CD16+CD56+ NK Cells** | 0.143 | 0.622 | -0.478 | 0.765 | 0.071 | 0.823 | -0.640 | 0.783 | -4.150 | 0.874 | -266.043 | 257.743 |
| **CD16+CD56++ NK Cells** | 0.006 | 0.756 | -0.034 | 0.046 | -0.004 | 0.811 | -0.045 | 0.036 | 0.870 | 0.608 | -14.756 | 16.496 |
| **CD16-CD56++ NK Cells** | -0.100 | 0.548 | -0.456 | 0.256 | -0.114 | 0.591 | -0.583 | 0.356 | 5.025 | 0.529 | -64.850 | 74.900 |
| **All CD3+ T cells** | -0.765 | 0.010 | -1.308 | -0.222 | -0.787 | 0.038 | -1.520 | -0.054 | 8.150 | 0.233 | -31.464 | 47.764 |
| **CD4+ T cells** | -0.013 | 0.918 | -0.275 | 0.249 | -0.018 | 0.887 | -0.302 | 0.266 | 9.650 | 0.167 | -23.362 | 42.662 |
| **CD8+ T cells** | -0.011 | 0.923 | -0.260 | 0.237 | 0.039 | 0.774 | -0.266 | 0.345 | -6.350 | 0.161 | -27.257 | 14.557 |
| **CD4+ Central Memory** | 0.000 | 0.999 | -0.195 | 0.195 | -0.096 | 0.355 | -0.320 | 0.129 | -0.638 | 0.422 | -6.965 | 5.690 |
| **CD4+ Naive** | 0.018 | 0.845 | -0.176 | 0.212 | 0.084 | 0.432 | -0.150 | 0.318 | 3.500 | 0.212 | -11.905 | 18.905 |
| **CD4+ Terminal Effector (TEMRA)** | -0.015 | 0.335 | -0.047 | 0.017 | 0.000 | 0.963 | -0.010 | 0.011 | -1.598 | 0.057 | -3.413 | 0.218 |
| **CD4+ Effector Memory** | -0.003 | 0.836 | -0.037 | 0.031 | 0.012 | 0.310 | -0.013 | 0.037 | -1.280 | 0.267 | -8.543 | 5.983 |
| **CD4+ PD-1+** | 0.007 | 0.165 | -0.003 | 0.017 | 0.007 | 0.256 | -0.007 | 0.021 | 0.000 | 1.000 | -1.871 | 1.871 |
| **CD4+ TIGIT +** | 0.004 | 0.624 | -0.012 | 0.019 | 0.000 | 0.976 | -0.020 | 0.020 | 0.031 | 0.933 | -3.694 | 3.756 |
| **CD4+ CD38hi** | -0.027 | 0.545 | -0.122 | 0.068 | -0.076 | 0.083 | -0.165 | 0.012 | 2.225 | 0.327 | -13.731 | 18.181 |
| **CD4+ Activated (HLADR+CD38hi)** | 0.000 | 0.969 | -0.012 | 0.013 | 0.002 | 0.750 | -0.013 | 0.018 | -0.278 | 0.348 | -2.423 | 1.868 |
| **CD4+ Tfh Cells** | 0.002 | 0.688 | -0.010 | 0.015 | 0.004 | 0.469 | -0.009 | 0.017 | 0.209 | 0.742 | -5.981 | 6.398 |
| **CD4+ Tfr Cells** | 0.069 | 0.265 | -0.060 | 0.197 | 0.025 | 0.734 | -0.139 | 0.189 | -0.828 | 0.465 | -10.236 | 8.581 |
| **Treg Cells (%CD25+CD127low)** | 0.028 | 0.327 | -0.032 | 0.088 | 0.036 | 0.296 | -0.038 | 0.111 | -1.588 | 0.036 | -2.743 | -0.432 |
| **Th17** | -0.011 | 0.749 | -0.082 | 0.061 | 0.022 | 0.167 | -0.011 | 0.054 | 0.760 | 0.852 | -40.059 | 41.579 |
| **Th1** | 0.155 | 0.295 | -0.156 | 0.466 | 0.109 | 0.574 | -0.321 | 0.539 | 2.425 | 0.079 | -1.426 | 6.276 |
| **Th2** | -0.142 | 0.352 | -0.462 | 0.179 | -0.131 | 0.507 | -0.564 | 0.302 | -3.600 | 0.558 | -58.619 | 51.419 |
| **CD8+ Central Memory** | -0.119 | 0.312 | -0.367 | 0.128 | -0.296 | 0.007* | -0.489 | -0.104 | 0.528 | 0.428 | -4.809 | 5.864 |
| **CD8+ Naive** | -0.038 | 0.778 | -0.324 | 0.249 | 0.095 | 0.536 | -0.244 | 0.433 | 0.650 | 0.619 | -11.454 | 12.754 |
| **CD8+ Terminal Effector (TEMRA)** | 0.024 | 0.323 | -0.027 | 0.074 | 0.055 | 0.007 | 0.020 | 0.090 | -1.335 | 0.336 | -11.239 | 8.569 |
| **CD8+ Effector Memory** | 0.133 | 0.049 | 0.000 | 0.265 | 0.146 | 0.106 | -0.039 | 0.330 | 0.153 | 0.844 | -7.605 | 7.910 |
| **CD8+ PD-1 +** | 0.003 | 0.624 | -0.010 | 0.016 | 0.006 | 0.345 | -0.008 | 0.020 | -0.129 | 0.819 | -5.713 | 5.456 |
| **CD8+TIGIT+** | 0.033 | 0.145 | -0.013 | 0.079 | 0.016 | 0.542 | -0.042 | 0.075 | -0.005 | 0.983 | -2.316 | 2.306 |

* asterix indicates analyses that are consistent between simple and age-adjusted multiple linear regression in supplementary table 7
