# Supplementary material for: Enzyme replacement therapy (ERT) combined with transient low-dose methotrexate (TLD-MTX) results in age- and disease-dependent immune profile changes in Infantile- vs. late-onset Pompe disease patients
Source: Front Immunol. 2026 Sep 4;17:1843379. doi: 10.3389/fimmu.2026.1843379 (PMC13585601; doi:10.3389/fimmu.2026.1843379)
Supplement: Supplementary file 1 [file Supplementaryfile1.zip › Supplementary Table 7.docx]

| **Immune markers** | **Multiple Linear Regression (Age-adjusted Glc_4_ vs immune markers)** | | | | | | | | | | | |
| --- | --- | --- | --- | --- | --- | --- | --- | --- | --- | --- | --- | --- |
|  | **All IOPD patients** | | | | **ERT+MTX-untreated IOPD patients** | | | | **ERT+MTX-treated IOPD patients** | | | |
|  | **Coefficient** | **P value** | **95% conf. low** | **95% conf. high** | **Coefficient** | **P value** | **95% conf. low** | **95% conf. high** | **Coefficient** | **P value** | **95% conf. low** | **95% conf. high** |
| **All CD14+ monocytes** | -0.372 | 0.249 | -1.048 | 0.305 | -0.544 | 0.437 | -2.104 | 1.015 | -0.883 | 0.272 | -2.975 | 1.209 |
| **Classical monocytes** | -0.285 | 0.005 | -0.463 | -0.108 | -0.153 | 0.372 | -0.531 | 0.226 | 1.340 | 0.250 | -1.658 | 4.337 |
| **CD16+ monocytes (non-classical + intermediate)** | 0.241 | 0.008 | 0.080 | 0.402 | 0.096 | 0.527 | -0.245 | 0.437 | -1.314 | 0.259 | -4.320 | 1.693 |
| **All Dendritic cells (DCs)** | 0.117 | 0.557 | -0.311 | 0.544 | 0.112 | 0.795 | -0.867 | 1.090 | 0.597 | 0.285 | -0.865 | 2.058 |
| **Myeloid Dendritic Cells (mDC; % DCs)** | 0.254 | 0.389 | -0.375 | 0.883 | -0.364 | 0.507 | -1.594 | 0.866 | 1.562 | 0.287 | -2.291 | 5.416 |
| **Plasmacytoid Dendritic Cells (pDC; % DCs)** | -0.056 | 0.656 | -0.327 | 0.216 | -0.118 | 0.660 | -0.729 | 0.492 | -0.732 | 0.610 | -4.839 | 3.376 |
| **All B cells** | 0.012 | 0.898 | -0.195 | 0.219 | 0.100 | 0.579 | -0.307 | 0.508 | -1.920 | 0.272 | -6.469 | 2.629 |
| **Memory B cells** | 0.016 | 0.504 | -0.036 | 0.069 | 0.065 | 0.006 | 0.025 | 0.104 | 1.654 | 0.422 | -4.025 | 7.334 |
| **Plasmablasts** | -0.098 | 0.385 | -0.338 | 0.142 | -0.170 | 0.393 | -0.612 | 0.272 | -0.133 | 0.780 | -1.519 | 1.253 |
| **Naïve B cells** | -0.081 | 0.227 | -0.220 | 0.059 | -0.182 | 0.100 | -0.408 | 0.045 | -2.178 | 0.536 | -12.131 | 7.774 |
| **Transitional B cells** | -0.225 | 0.305 | -0.689 | 0.239 | -0.538 | 0.192 | -1.420 | 0.344 | -0.033 | 0.794 | -0.404 | 0.338 |
| **Unswitched Memory B cells** | -0.008 | 0.407 | -0.030 | 0.013 | -0.015 | 0.272 | -0.043 | 0.014 | 0.598 | 0.628 | -2.944 | 4.140 |
| **NK Cells** | 0.042 | 0.420 | -0.069 | 0.153 | -0.062 | 0.552 | -0.294 | 0.171 | -0.433 | 0.694 | -3.613 | 2.748 |
| **CD16+CD56+ NK Cells** | 0.117 | 0.689 | -0.517 | 0.751 | -0.203 | 0.707 | -1.428 | 1.022 | 6.307 | 0.067 | -0.811 | 13.426 |
| **CD16+CD56++ NK Cells** | 0.005 | 0.782 | -0.037 | 0.048 | -0.016 | 0.617 | -0.087 | 0.055 | 0.242 | 0.802 | -2.565 | 3.048 |
| **CD16-CD56++ NK Cells** | -0.087 | 0.608 | -0.455 | 0.280 | -0.031 | 0.932 | -0.859 | 0.798 | 0.218 | 0.914 | -5.725 | 6.162 |
| **All CD3+ T cells** | 0.000 | 0.917 | -0.004 | 0.003 | -0.108 | 0.814 | -1.158 | 0.942 | 2.775 | 0.396 | -6.174 | 11.724 |
| **CD4+ T cells** | -0.002 | 0.990 | -0.269 | 0.266 | 0.145 | 0.488 | -0.324 | 0.614 | 0.465 | 0.698 | -2.990 | 3.920 |
| **CD8+ T cells** | -0.016 | 0.892 | -0.278 | 0.246 | 0.272 | 0.217 | -0.202 | 0.745 | 0.365 | 0.702 | -2.395 | 3.124 |
| **CD4+ Central Memory** | 0.006 | 0.952 | -0.198 | 0.210 | -0.332 | 0.032 | -0.627 | -0.038 | 0.691 | 0.893 | -14.405 | 15.786 |
| **CD4+ Naive** | 0.018 | 0.849 | -0.189 | 0.225 | 0.341 | 0.029 | 0.045 | 0.637 | -0.707 | 0.903 | -17.662 | 16.249 |
| **CD4+ Terminal Effector (TEMRA)** | -0.017 | 0.214 | -0.046 | 0.012 | 0.004 | 0.638 | -0.014 | 0.022 | -0.242 | 0.765 | -2.589 | 2.106 |
| **CD4+ Effector Memory** | -0.007 | 0.544 | -0.031 | 0.018 | -0.012 | 0.458 | -0.048 | 0.024 | 0.273 | 0.853 | -4.030 | 4.576 |
| **CD4+ PD-1+** | 0.007 | 0.192 | -0.004 | 0.018 | 0.016 | 0.139 | -0.007 | 0.039 | -0.087 | 0.109 | -0.210 | 0.036 |
| **CD4+ TIGIT +** | 0.004 | 0.648 | -0.013 | 0.020 | -0.007 | 0.636 | -0.042 | 0.028 | -0.085 | 0.497 | -0.434 | 0.264 |
| **CD4+ CD38hi** | -0.016 | 0.560 | -0.074 | 0.042 | 0.037 | 0.362 | -0.053 | 0.128 | 0.286 | 0.870 | -4.840 | 5.412 |
| **CD4+ Activated (HLADR+CD38hi)** | 0.000 | 0.960 | -0.013 | 0.012 | 0.004 | 0.714 | -0.023 | 0.032 | -0.203 | 0.068 | -0.434 | 0.028 |
| **CD4+ Tfh Cells** | 0.002 | 0.781 | -0.011 | 0.014 | 0.002 | 0.815 | -0.020 | 0.025 | 0.160 | 0.837 | -2.109 | 2.429 |
| **CD4+ Tfr Cells** | 0.072 | 0.258 | -0.062 | 0.207 | -0.080 | 0.499 | -0.346 | 0.186 | -0.602 | 0.043 | -1.171 | -0.034 |
| **Treg Cells (%CD25+CD127low)** | 0.026 | 0.374 | -0.036 | 0.089 | 0.021 | 0.722 | -0.111 | 0.152 | -0.153 | 0.487 | -0.767 | 0.461 |
| **Th17** | -0.019 | 0.369 | -0.064 | 0.026 | 0.010 | 0.688 | -0.047 | 0.067 | 0.418 | 0.630 | -2.066 | 2.901 |
| **Th1** | 0.160 | 0.307 | -0.171 | 0.490 | 0.033 | 0.920 | -0.725 | 0.792 | -1.684 | 0.479 | -8.329 | 4.961 |
| **Th2** | -0.136 | 0.393 | -0.475 | 0.203 | -0.032 | 0.923 | -0.793 | 0.729 | 1.200 | 0.734 | -9.049 | 11.449 |
| **CD8+ Central Memory** | -0.100 | 0.339 | -0.322 | 0.122 | -0.445 | 0.009 | -0.742 | -0.148 | 0.144 | 0.677 | -0.857 | 1.145 |
| **CD8+ Naive** | -0.043 | 0.756 | -0.346 | 0.259 | 0.492 | 0.022 | 0.095 | 0.890 | -3.044 | 0.198 | -8.920 | 2.832 |
| **CD8+ Terminal Effector (TEMRA)** | 0.018 | 0.264 | -0.016 | 0.052 | 0.043 | 0.137 | -0.018 | 0.104 | 1.075 | 0.756 | -8.989 | 11.139 |
| **CD8+ Effector Memory** | 0.125 | 0.055 | -0.003 | 0.253 | -0.092 | 0.284 | -0.279 | 0.095 | 1.837 | 0.643 | -9.543 | 13.216 |
| **CD8+ PD-1 +** | 0.002 | 0.702 | -0.010 | 0.015 | 0.019 | 0.059 | -0.001 | 0.038 | 0.073 | 0.920 | -2.052 | 2.198 |
| **CD8+TIGIT+** | 0.034 | 0.138 | -0.013 | 0.082 | 0.009 | 0.837 | -0.094 | 0.113 | -0.467 | 0.777 | -5.279 | 4.344 |
