# Supplementary material for: Enzyme replacement therapy (ERT) combined with transient low-dose methotrexate (TLD-MTX) results in age- and disease-dependent immune profile changes in Infantile- vs. late-onset Pompe disease patients
Source: Front Immunol. 2026 Sep 4;17:1843379. doi: 10.3389/fimmu.2026.1843379 (PMC13585601; doi:10.3389/fimmu.2026.1843379)
Supplement: Supplementary file 1 [file Supplementaryfile1.zip › Supplementary Table 8.docx]

| **Immune Markers** | **Simple Linear Regression (age vs immune markers)** | | | | | | | | **Simple Linear Regression (Glc_4_ vs immune markers)** | | | | | | | |
| --- | --- | --- | --- | --- | --- | --- | --- | --- | --- | --- | --- | --- | --- | --- | --- | --- |
|  | **Pre-ERT+MTX LOPD patients** | | | | **Post-ERT+MTX LOPD patients** | | | | **Pre-ERT+MTX LOPD patients** | | | | **Post-ERT+MTX LOPD patients** | | | |
|  | **Coefficient** | **P value** | **95% conf. low** | **95% conf. high** | **Coefficient** | **P value** | **95% conf. low** | **95% conf. high** | **Coefficient** | **P value** | **95% conf. low** | **95% conf. high** | **Coefficient** | **P value** | **95% conf. low** | **95% conf. high** |
| **All CD14+ monocytes** | 0.009 | 0.078 | 0.110 | 0.917 | 0.047 | 0.614 | -0.192 | 0.286 | -0.338 | 0.430 | -1.407 | 0.732 | -0.406 | 0.653 | -2.724 | 1.913 |
| **Classical monocytes** | -0.075 | 0.114 | -0.660 | 0.543 | -0.254 | 0.016 | -0.430 | -0.077 | 0.787 | 0.201 | -0.643 | 2.218 | 2.540 | 0.007* | 1.132 | 3.947 |
| **CD16+ monocytes (non-classical + intermediate)** | 0.075 | 0.113 | 0.660 | 0.543 | 0.251 | 0.014 | 0.083 | 0.419 | -0.777 | 0.206 | -2.205 | 0.651 | -2.493 | 0.008* | -3.888 | -1.098 |
| **All Dendritic cells (DCs)** | -0.015 | 0.054 | -0.280 | 0.793 | -0.039 | 0.232 | -0.117 | 0.038 | 0.269 | 0.360 | -0.453 | 0.990 | 0.390 | 0.216 | -0.348 | 1.129 |
| **Myeloid Dendritic Cells (mDC; % DCs)** | 0.441 | 0.141 | 3.130 | 0.035 | 0.426 | 0.149 | -0.237 | 1.088 | -1.408 | 0.316 | -4.818 | 2.003 | -3.042 | 0.320 | -10.494 | 4.409 |
| **Plasmacytoid Dendritic Cells (pDC; % DCs)** | -0.217 | 0.127 | -1.720 | 0.161 | -0.215 | 0.447 | -0.926 | 0.495 | 0.708 | 0.442 | -1.600 | 3.016 | 0.329 | 0.908 | -7.091 | 7.749 |
| **B cells** | 0.116 | 0.169 | 0.690 | 0.531 | 0.041 | 0.776 | -0.336 | 0.418 | -1.161 | 0.207 | -3.303 | 0.980 | -0.145 | 0.918 | -3.817 | 3.528 |
| **Memory B cells** | -0.009 | 0.189 | -0.050 | 0.963 | 0.020 | 0.943 | -0.703 | 0.743 | 0.603 | 0.571 | -2.109 | 3.315 | -1.084 | 0.682 | -7.899 | 5.731 |
| **Plasmablasts** | -0.005 | 0.041 | -0.130 | 0.905 | 0.079 | 0.617 | -0.324 | 0.481 | -0.022 | 0.926 | -0.643 | 0.598 | -0.848 | 0.573 | -4.693 | 2.997 |
| **Naïve B cells** | -0.149 | 0.314 | -0.470 | 0.661 | -0.099 | 0.870 | -1.682 | 1.483 | -0.086 | 0.963 | -4.940 | 4.768 | 3.041 | 0.597 | -11.687 | 17.770 |
| **Transitional B cells** | -0.014 | 0.011 | -1.290 | 0.268 | 0.023 | 0.715 | -0.142 | 0.188 | 0.051 | 0.484 | -0.133 | 0.235 | -0.181 | 0.770 | -1.785 | 1.423 |
| **Unswitched Memory B cells** | 0.071 | 0.109 | 0.660 | 0.548 | 0.191 | 0.026 | 0.038 | 0.344 | -0.110 | 0.867 | -1.822 | 1.602 | -1.566 | 0.095 | -3.559 | 0.427 |
| **NK Cells** | 0.406 | 0.096 | 4.230 | 0.013 | 0.514 | 0.020 | 0.134 | 0.894 | -1.933 | 0.059 | -3.981 | 0.115 | -4.518 | 0.054 | -9.156 | 0.120 |
| **CD16+CD56+ NK Cells** | 0.231 | 0.398 | 0.580 | 0.593 | 0.758 | 0.096 | -0.212 | 1.727 | 1.126 | 0.632 | -4.913 | 7.165 | -4.422 | 0.377 | -16.777 | 7.933 |
| **CD16+CD56++ NK Cells** | -0.070 | 0.083 | -0.850 | 0.445 | 0.015 | 0.366 | -0.026 | 0.056 | 0.392 | 0.432 | -0.853 | 1.637 | -0.143 | 0.370 | -0.536 | 0.251 |
| **CD16-CD56++ NK Cells** | -0.236 | 0.174 | -1.350 | 0.248 | -0.186 | 0.055 | -0.378 | 0.006 | 1.112 | 0.327 | -1.653 | 3.877 | 1.342 | 0.207 | -1.138 | 3.822 |
| **All CD3+ T cells** | -0.542 | 0.342 | -1.580 | 0.189 | -0.628 | 0.039 | -1.203 | -0.052 | 3.925 | 0.041* | 0.257 | 7.593 | 5.741 | 0.061 | -0.418 | 11.899 |
| **CD4+ T cells** | 0.472 | 0.104 | 4.520 | 0.011 | 0.418 | 0.008* | 0.185 | 0.652 | -1.917 | 0.132 | -4.736 | 0.901 | -3.758 | 0.026* | -6.796 | -0.720 |
| **CD8+ T cells** | -0.378 | 0.083 | -4.540 | 0.010 | -0.354 | 0.002* | -0.486 | -0.222 | 1.787 | 0.057 | -0.080 | 3.654 | 3.248 | 0.01* | 1.309 | 5.187 |
| **CD4+ Central Memory** | 0.493 | 0.444 | 1.110 | 0.329 | 0.476 | 0.302 | -0.640 | 1.593 | -1.933 | 0.494 | -9.078 | 5.211 | -3.882 | 0.394 | -15.181 | 7.417 |
| **CD4+ Naive** | -0.653 | 0.498 | -1.310 | 0.260 | -0.525 | 0.248 | -1.604 | 0.553 | 2.634 | 0.422 | -5.542 | 10.810 | 3.790 | 0.408 | -7.614 | 15.193 |
| **CD4+ Terminal Effector (TEMRA)** | 0.036 | 0.070 | 0.520 | 0.630 | 0.009 | 0.941 | -0.300 | 0.318 | -0.243 | 0.550 | -1.277 | 0.791 | -0.077 | 0.946 | -3.057 | 2.902 |
| **CD4+ Effector Memory** | 0.126 | 0.127 | 1.000 | 0.376 | 0.040 | 0.723 | -0.249 | 0.328 | -0.464 | 0.561 | -2.496 | 1.569 | 0.185 | 0.865 | -2.639 | 3.008 |
| **CD4+ PD-1+** | 0.004 | 0.006 | 0.650 | 0.554 | 0.021 | 0.010 | 0.008 | 0.033 | -0.046 | 0.129 | -0.114 | 0.021 | -0.162 | 0.090 | -0.364 | 0.040 |
| **CD4+ TIGIT +** | 0.027 | 0.011 | 2.380 | 0.076 | 0.024 | 0.111 | -0.009 | 0.058 | -0.146 | 0.071 | -0.311 | 0.020 | -0.241 | 0.100 | -0.555 | 0.073 |
| **CD4+ CD38hi** | -0.770 | 0.151 | -5.100 | 0.007 | -0.353 | 0.150 | -0.904 | 0.199 | 3.487 | 0.068 | -0.420 | 7.393 | 2.389 | 0.352 | -3.906 | 8.683 |
| **CD4+ Activated (HLADR+CD38hi)** | 0.001 | 0.013 | 0.080 | 0.942 | 0.003 | 0.745 | -0.023 | 0.029 | -0.073 | 0.283 | -0.238 | 0.091 | -0.079 | 0.397 | -0.310 | 0.152 |
| **CD4+ Tfh Cells** | -0.037 | 0.067 | -0.550 | 0.609 | 0.033 | 0.624 | -0.140 | 0.206 | 0.218 | 0.578 | -0.783 | 1.219 | -0.074 | 0.911 | -1.793 | 1.646 |
| **CD4+ Tfr Cells** | 0.005 | 0.036 | 0.120 | 0.907 | 0.027 | 0.097 | -0.008 | 0.062 | -0.225 | 0.240 | -0.677 | 0.227 | -0.224 | 0.182 | -0.610 | 0.162 |
| **Treg Cells (%CD25+CD127low)** | 0.006 | 0.020 | 0.310 | 0.770 | -0.019 | 0.335 | -0.067 | 0.029 | -0.079 | 0.477 | -0.359 | 0.201 | 0.234 | 0.199 | -0.188 | 0.655 |
| **Th17** | -0.107 | 0.076 | -1.410 | 0.231 | -0.065 | 0.553 | -0.347 | 0.216 | 0.614 | 0.200 | -0.497 | 1.726 | 1.143 | 0.251 | -1.226 | 3.512 |
| **Th1** | 0.184 | 0.215 | 0.860 | 0.441 | 0.092 | 0.705 | -0.537 | 0.721 | -1.380 | 0.263 | -4.323 | 1.562 | -1.750 | 0.442 | -7.448 | 3.948 |
| **Th2** | -0.031 | 0.307 | -0.100 | 0.925 | -0.011 | 0.972 | -0.825 | 0.803 | 0.543 | 0.758 | -4.019 | 5.104 | 0.327 | 0.913 | -7.515 | 8.168 |
| **CD8+ Central Memory** | 0.044 | 0.030 | 1.440 | 0.224 | 0.083 | 0.115 | -0.032 | 0.197 | -0.143 | 0.492 | -0.669 | 0.383 | -0.637 | 0.241 | -1.924 | 0.650 |
| **CD8+ Naive** | -0.992 | 0.238 | -4.170 | 0.014 | -0.987 | 0.033 | -1.848 | -0.126 | 3.331 | 0.249 | -3.526 | 10.187 | 7.315 | 0.162 | -4.545 | 19.174 |
| **CD8+ Terminal Effector (TEMRA)** | 0.438 | 0.300 | 1.460 | 0.219 | 0.331 | 0.408 | -0.664 | 1.325 | -1.562 | 0.447 | -6.714 | 3.591 | -2.624 | 0.503 | -12.543 | 7.295 |
| **CD8+ Effector Memory** | 0.511 | 0.348 | 1.470 | 0.215 | 0.573 | 0.225 | -0.536 | 1.682 | -1.626 | 0.499 | -7.710 | 4.458 | -4.052 | 0.398 | -15.955 | 7.852 |
| **CD8+ PD-1 +** | 0.132 | 0.062 | 2.110 | 0.102 | 0.210 | 0.094 | -0.056 | 0.477 | -0.555 | 0.243 | -1.681 | 0.571 | -1.716 | 0.185 | -4.696 | 1.263 |
| **CD8+TIGIT+** | 0.365 | 0.143 | 2.550 | 0.064 | 0.444 | 0.079 | -0.082 | 0.969 | -1.763 | 0.120 | -4.245 | 0.718 | -4.237 | 0.084 | -9.378 | 0.905 |
