# Supplementary material for: Enzyme replacement therapy (ERT) combined with transient low-dose methotrexate (TLD-MTX) results in age- and disease-dependent immune profile changes in Infantile- vs. late-onset Pompe disease patients
Source: Front Immunol. 2026 Sep 4;17:1843379. doi: 10.3389/fimmu.2026.1843379 (PMC13585601; doi:10.3389/fimmu.2026.1843379)
Supplement: Supplementary file 1 [file Supplementaryfile1.zip › Supplementary Table 9.docx]

| **Immune markers** | **Multiple Linear Regression (Age-adjusted Glc_4_ vs immune markers)** | | | | | | | |
| --- | --- | --- | --- | --- | --- | --- | --- | --- |
|  | **Pre-ERT+MTX LOPD patients** | | | | **Post-ERT+MTX LOPD patients** | | | |
|  | **Coefficient** | **P value** | **95% conf. low** | **95% conf. high** | **Coefficient** | **P value** | **95% conf. low** | **95% conf. high** |
| **All CD14+ monocytes** | -0.883 | 0.272 | -2.975 | 1.209 | -0.053 | 0.980 | -6.131 | 6.025 |
| **Classical monocytes** | 1.340 | 0.250 | -1.658 | 4.337 | 1.686 | 0.197 | -1.560 | 4.932 |
| **CD16+ monocytes (non-classical + intermediate)** | -1.314 | 0.259 | -4.320 | 1.693 | -1.584 | 0.207 | -4.725 | 1.556 |
| **All Dendritic cells (DCs)** | 0.597 | 0.285 | -0.865 | 2.058 | 0.244 | 0.713 | -1.682 | 2.171 |
| **Myeloid Dendritic Cells (mDC; % DCs)** | 1.562 | 0.287 | -2.291 | 5.416 | 2.041 | 0.720 | -14.423 | 18.504 |
| **Plasmacytoid Dendritic Cells (pDC; % DCs)** | -0.732 | 0.610 | -4.839 | 3.376 | -5.853 | 0.291 | -20.410 | 8.703 |
| **B cells** | -1.920 | 0.272 | -6.469 | 2.629 | 0.799 | 0.806 | -8.694 | 10.291 |
| **Memory B cells** | 1.654 | 0.422 | -4.025 | 7.334 | -3.663 | 0.545 | -20.814 | 13.488 |
| **Plasmablasts** | -0.133 | 0.780 | -1.519 | 1.253 | -0.767 | 0.825 | -10.923 | 9.389 |
| **Naïve B cells** | -2.178 | 0.536 | -12.131 | 7.774 | 8.816 | 0.503 | -28.109 | 45.741 |
| **Transitional B cells** | -0.033 | 0.794 | -0.404 | 0.338 | 0.053 | 0.970 | -4.155 | 4.261 |
| **Unswitched Memory B cells** | 0.598 | 0.628 | -2.944 | 4.140 | 0.102 | 0.939 | -3.792 | 3.997 |
| **NK Cells** | -0.433 | 0.694 | -3.613 | 2.748 | -0.901 | 0.783 | -10.447 | 8.645 |
| **CD16+CD56+ NK Cells** | 6.307 | 0.067 | -0.811 | 13.426 | 7.579 | 0.322 | -12.818 | 27.976 |
| **CD16+CD56++ NK Cells** | 0.242 | 0.802 | -2.565 | 3.048 | -0.073 | 0.837 | -1.101 | 0.956 |
| **CD16-CD56++ NK Cells** | 0.218 | 0.914 | -5.725 | 6.162 | -0.837 | 0.606 | -5.476 | 3.803 |
| **All CD3+ T cells** | 4.529 | 0.178 | -3.685 | 12.743 | 1.997 | 0.685 | -12.198 | 16.191 |
| **CD4+ T cells** | 0.465 | 0.698 | -2.990 | 3.920 | -1.056 | 0.591 | -6.670 | 4.557 |
| **CD8+ T cells** | 0.365 | 0.702 | -2.395 | 3.124 | 1.167 | 0.248 | -1.428 | 3.762 |
| **CD4+ Central Memory** | 0.691 | 0.893 | -14.405 | 15.786 | 0.381 | 0.969 | -28.063 | 28.825 |
| **CD4+ Naive** | -0.707 | 0.903 | -17.662 | 16.249 | -2.371 | 0.799 | -29.509 | 24.768 |
| **CD4+ Terminal Effector (TEMRA)** | -0.242 | 0.765 | -2.589 | 2.106 | -0.016 | 0.995 | -7.886 | 7.855 |
| **CD4+ Effector Memory** | 0.273 | 0.853 | -4.030 | 4.576 | 2.050 | 0.378 | -4.271 | 8.371 |
| **CD4+ PD-1+** | -0.087 | 0.109 | -0.210 | 0.036 | 0.044 | 0.675 | -0.260 | 0.349 |
| **CD4+ TIGIT +** | -0.085 | 0.497 | -0.434 | 0.264 | -0.146 | 0.605 | -0.952 | 0.660 |
| **CD4+ CD38hi** | 0.286 | 0.870 | -4.840 | 5.412 | -2.215 | 0.637 | -15.667 | 11.238 |
| **CD4+ Activated (HLADR+CD38hi)** | -0.203 | 0.068 | -0.434 | 0.028 | -0.205 | 0.320 | -0.753 | 0.343 |
| **CD4+ Tfh Cells** | 0.160 | 0.837 | -2.109 | 2.429 | 0.802 | 0.582 | -3.344 | 4.947 |
| **CD4+ Tfr Cells** | -0.602 | 0.043 | -1.171 | -0.034 | 0.010 | 0.974 | -0.880 | 0.899 |
| **Treg Cells (%CD25+CD127low)** | -0.153 | 0.487 | -0.767 | 0.461 | 0.297 | 0.456 | -0.808 | 1.402 |
| **Th17** | 0.418 | 0.630 | -2.066 | 2.901 | 2.378 | 0.275 | -3.304 | 8.061 |
| **Th1** | -1.684 | 0.479 | -8.329 | 4.961 | -3.907 | 0.450 | -18.246 | 10.432 |
| **Th2** | 1.200 | 0.734 | -9.049 | 11.449 | 0.942 | 0.894 | -19.733 | 21.616 |
| **CD8+ Central Memory** | 0.144 | 0.677 | -0.857 | 1.145 | 0.209 | 0.833 | -2.678 | 3.095 |
| **CD8+ Naive** | -3.044 | 0.198 | -8.920 | 2.832 | -3.696 | 0.612 | -24.556 | 17.165 |
| **CD8+ Terminal Effector (TEMRA)** | 1.075 | 0.756 | -8.989 | 11.139 | 0.554 | 0.949 | -24.765 | 25.874 |
| **CD8+ Effector Memory** | 1.837 | 0.643 | -9.543 | 13.216 | 2.921 | 0.760 | -24.822 | 30.664 |
| **CD8+ PD-1 +** | 0.073 | 0.920 | -2.052 | 2.198 | 0.161 | 0.945 | -6.626 | 6.948 |
| **CD8+TIGIT+** | -0.467 | 0.777 | -5.279 | 4.344 | -2.116 | 0.636 | -14.931 | 10.699 |
